# Supplementary figures and images for: Variation in antibiotic prescription rates in febrile children presenting to emergency departments across Europe (MOFICHE): A multicentre observational study
Source: PLoS Med. 2020 Aug 19;17(8):e1003208. doi: 10.1371/journal.pmed.1003208 (PMC7444592; doi:10.1371/journal.pmed.1003208)

Duration for prescribed antibiotic (n=9391)

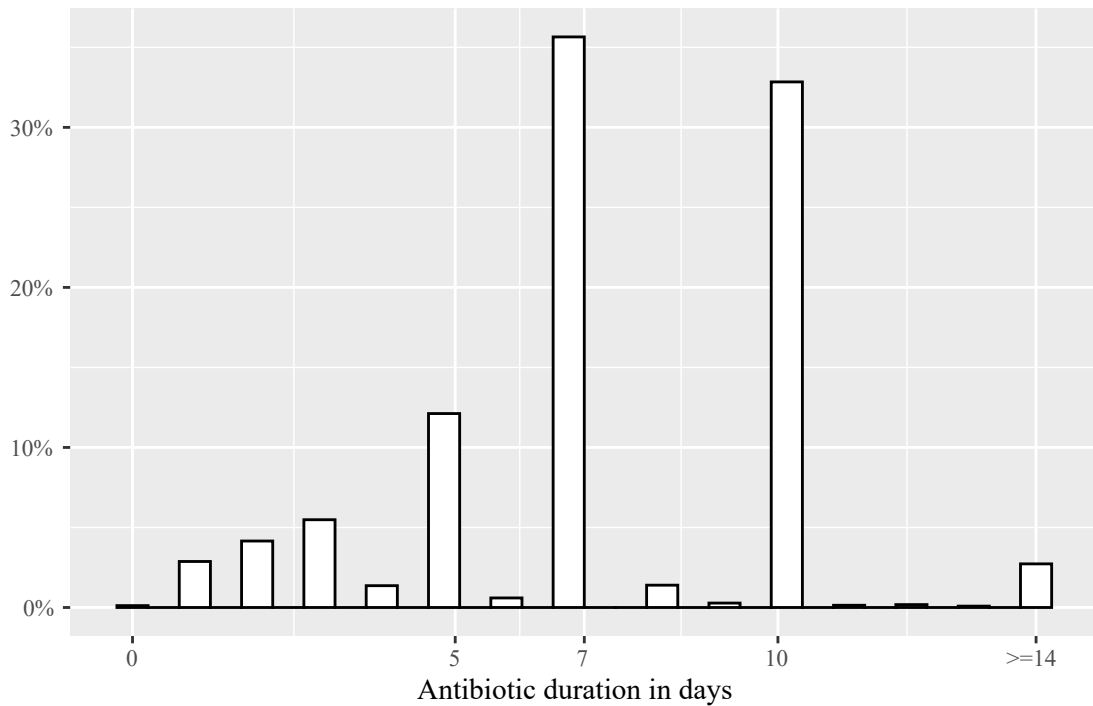

Supplement: S1 Fig — (PDF) [file pmed.1003208.s001.pdf]
