# Supplementary material for: Variation in antibiotic prescription rates in febrile children presenting to emergency departments across Europe (MOFICHE): A multicentre observational study
Source: PLoS Med. 2020 Aug 19;17(8):e1003208. doi: 10.1371/journal.pmed.1003208 (PMC7444592; doi:10.1371/journal.pmed.1003208)

**S2 Fig: Range of antibiotic prescriptions and broad−spectrum prescriptions per emergency department (ED) for viral, bacterial, and unknown bacterial/viral infections. (A) antibiotic prescriptions; (B) broad-spectrum prescriptions.**

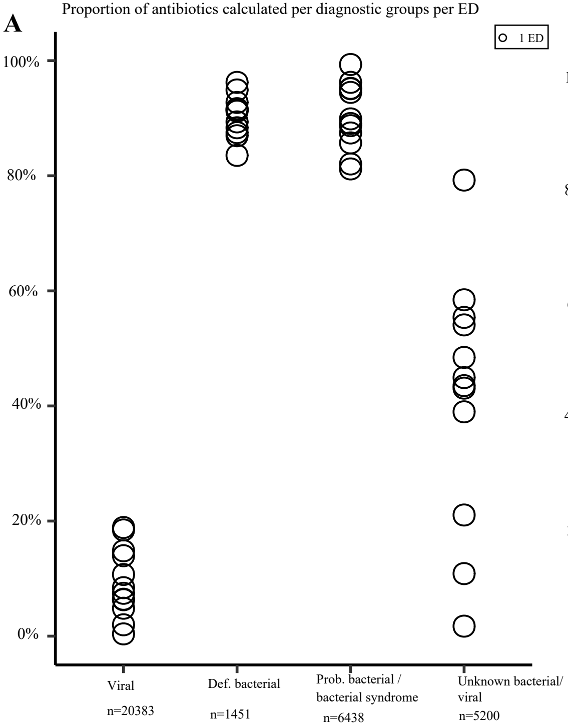


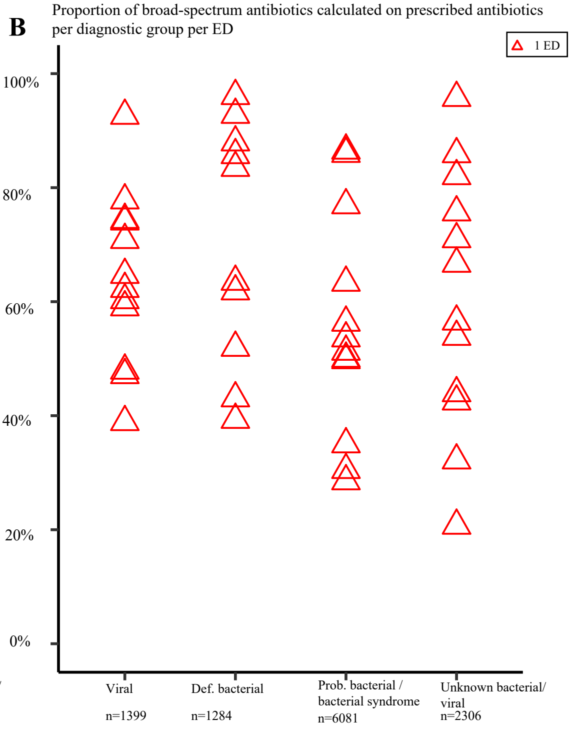

Supplement: S2 Fig — (A) antibiotic prescriptions; (B) broad-spectrum prescriptions. (PDF) [file pmed.1003208.s002.pdf]
