# Supplementary material for: Variation in antibiotic prescription rates in febrile children presenting to emergency departments across Europe (MOFICHE): A multicentre observational study
Source: PLoS Med. 2020 Aug 19;17(8):e1003208. doi: 10.1371/journal.pmed.1003208 (PMC7444592; doi:10.1371/journal.pmed.1003208)
Supplement: S1 Text — (PDF) [file pmed.1003208.s004.pdf]

STROBE Statement—checklist of items that should be included in reports of observational studies

	Item No.	Recommendation	Section	Relevant text from manuscript
**Title and abstract**	1	(*a*) Indicate the study's design with a commonly used term in the title or the abstract	Title	*The study design, is stated in the title.*
		(*b*) Provide in the abstract an informative and balanced summary of what was done and what was found	Abstract	*The abstract is written according to the PLOS guidelines with all required information.*
Background/rationale	2	Explain the scientific background and rationale for the investigation being reported	Introduction, first three paragraphs	The background and rationale are described in the first three paragraphs of the introduction.
Objectives	3	State specific objectives, including any prespecified hypotheses	Last paragraph of the introduction	The objective is stated in the last paragraph of the introduction.
Study design	4	Present key elements of study design early in the paper	Last paragraph of the introduction, First paragraph of Methods ('study design')	Key elements of the study design are presented in the first paragraph of the methods, and in detail explained in the consequent paragraphs.
Setting	5	Describe the setting, locations, and relevant dates, including periods of recruitment, exposure, follow-up, and data collection	Methods: 'Study population and setting' , Supplemental file 4	The setting, location and relevant dates are described in paragraph 'study population and setting', in the methods section and in supplemental 4.

Participants	6	(*a*) *Cohort study*—Give the eligibility criteria, and the sources and methods of selection of participants. Describe methods of follow-up<br>*Case-control study*—Give the eligibility criteria, and the sources and methods of case ascertainment and control selection. Give the rationale for the choice of cases and controls<br>*Cross-sectional study*—Give the eligibility criteria, and the sources and methods of selection of participants	Study population and setting, data collection	Described in section 'Study population and setting', 'Data collection' and 'Data analysis' in the methods section.
		(*b*) *Cohort study*—For matched studies, give matching criteria and number of exposed and unexposed<br>*Case-control study*—For matched studies, give matching criteria and the number of controls per case		Not applicable.
Variables	7	Clearly define all outcomes, exposures, predictors, potential confounders, and effect modifiers. Give diagnostic criteria, if applicable	Methods: Data collection, Antibiotic classification, Fig 1, supplemental 4 and 5	Described in 'Data collection', 'Antibiotic classification', 'Outcomes', 'Data analysis' and Fig 1in the methods section. Details are provided in supplemental 4 and supplemental 5.
Data sources/ measurement	8*	For each variable of interest, give sources of data and details of methods of assessment (measurement). Describe comparability of assessment methods if there is more than one group	Methods: Data collection, Antibiotic classification and Data analysis	Described in 'Data collection', 'Antibiotic classification' and 'Data analysis'.
Bias	9	Describe any efforts to address potential sources of bias	Methods: data collection, data analysis. Table 4. Supplemental 7.	Information bias was limited by entry guidelines for the eCRF, standardized data collection, training modules, feedback and teleconferences. Described in 'Data collection'. We adjusted for differences in population by adjusting for general

				characteristics, disease severity, diagnostics, focus and cause of infection.
Study size	10	Explain how the study size was arrived at	Methods: sample size	Described in methods – sample size.

Continued on next page

Quantitative variables	11	Explain how quantitative variables were handled in the analyses. If applicable, describe which groupings were chosen and why	Methods: data analysis, Table 1, Supplemental 7	Described in 'Data analysis'. Linearity of continuous variables was tested using restricted cubic splines. Details of the adjusted model are described in supplemental 7.
Statistical methods	12	(*a*) Describe all statistical methods, including those used to control for confounding	Methods: data analysis	Described in 'Data analysis'.
		(*b*) Describe any methods used to examine subgroups and interactions	Methods: data analysis	Described in 'Data analysis'.
		(*c*) Explain how missing data were addressed	Methods: data analysis	Described in 'Data analysis'.
		(*d*) *Cohort study*—If applicable, explain how loss to follow-up was addressed<br>*Case-control study*—If applicable, explain how matching of cases and controls was addressed<br>*Cross-sectional study*—If applicable, describe analytical methods taking account of sampling strategy		Not applicable.
		(*e*) Describe any sensitivity analyses		Not applicable.
Participants	13*	(a) Report numbers of individuals at each stage of study—eg numbers potentially eligible, examined for eligibility, confirmed eligible, included in the study, completing follow-up, and analysed	Results: first paragraph	The patient flow is described in the first paragraph of the results section. All tables and figures included the number of patients that were analysed.
		(b) Give reasons for non-participation at each stage		Not applicable.
		(c) Consider use of a flow diagram		-
Descriptive data	14*	(a) Give characteristics of study participants (eg demographic, clinical, social) and information on exposures and potential confounders	Results: first paragraph, second paragraph, table 2, table 3	Characteristics of the study population are described in table 2 and table 3.
		(b) Indicate number of participants with missing data for each variable of interest	Table 2 and Table 3	Missing data for each variable are described in table 1 and table 2.
		(c) *Cohort study*—Summarise follow-up time (eg, average and total amount)		Not applicable.
Outcome data	15*	*Cohort study*—Report numbers of outcome events or summary measures over time	Table 2	The number of patients with the outcome are reported in table 2.

		Case-control study—Report numbers in each exposure category, or summary measures of exposure		
		Cross-sectional study—Report numbers of outcome events or summary measures		
Main results	16	(*a*) Give unadjusted estimates and, if applicable, confounder-adjusted estimates and their precision (eg, 95% confidence interval). Make clear which confounders were adjusted for and why they were included	Results: Paragraph 3 and paragraph 4, Fig 2, Fig. 3, Fig. 4, Fig. 5, appendix 11, appendix 12, appendix 13, appendix 14	Unadjusted prescription rates and standardized prescription rates (adjusted for general characteristics, disease severity, diagnostics and focus and cause of infection) and their precision are provided in the results section, Fig. 2, Fig. 3, Fig. 4, Fig. 5, Supplemental 11.
		(*b*) Report category boundaries when continuous variables were categorized	Methods: data analysis.	Described in section 'Data analysis' in method section.
		(*c*) If relevant, consider translating estimates of relative risk into absolute risk for a meaningful time period		Not applicable.

Continued on next page

Other analyses	17	Report other analyses done—eg analyses of subgroups and interactions, and sensitivity analyses	Methods: data analysis, Results: paragraph 4, 5, 6, 7	Pre-specified subgroup analyses were performed and described in the section 'Data analysis' of the methods section and in the results section. Appropriateness of antibiotic prescription was evaluated in different subgroups.
**Discussion**				
Key results	18	Summarise key results with reference to study objectives	Disucssion: first paragraph	Key results are stated in the first paragraph and discussed in the subsequent paragraphs of the discussion section.
Limitations	19	Discuss limitations of the study, taking into account sources of potential bias or imprecision. Discuss both direction and magnitude of any potential bias	Discussion: paragraph 5	Limitations of the study are addressed in the paragraph of the discussion, starting with "This study has some limitations…"
Interpretation	20	Give a cautious overall interpretation of results considering objectives, limitations, multiplicity of analyses, results from similar studies, and other relevant evidence	Discussion: paragraph 2, 3, 4, 5, 6, 7, 8, 9	Interpretation of the results is described in the discussion.
Generalisability	21	Discuss the generalisability (external validity) of the study results	Discussion: paragraph 5	Generalizability of the results is discussed in the paragraph discussing the limitations.
**Other information**				
Funding	22	Give the source of funding and the role of the funders for the present study and, if applicable, for the original study on which the present article is based		Title page.

*Give information separately for cases and controls in case-control studies and, if applicable, for exposed and unexposed groups in cohort and cross-sectional studies.

Note: An Explanation and Elaboration article discusses each checklist item and gives methodological background and published examples of transparent reporting. The STROBE checklist is best used in conjunction with this article (freely available on the Web sites of PLoS Medicine at http://www.plosmedicine.org/, Annals of Internal Medicine at http://www.annals.org/, and Epidemiology at http://www.epidem.com/). Information on the STROBE Initiative is available at www.strobe-statement.org.
