# Supplementary material for: Variation in antibiotic prescription rates in febrile children presenting to emergency departments across Europe (MOFICHE): A multicentre observational study
Source: PLoS Med. 2020 Aug 19;17(8):e1003208. doi: 10.1371/journal.pmed.1003208 (PMC7444592; doi:10.1371/journal.pmed.1003208)
Supplement: S2 Text — (PDF) [file pmed.1003208.s005.pdf]

# Supplemental 2: Statistical Analysis Plan

Diversity in antibiotic prescription in febrile children presenting to Emergency Departments in Europe

## Addition 14 Feb 2020

### Planning

First, pilot analyses were performed halfway of data collection (November 2017) and final analyses upon the complete data set were performed in December/January 2018.

The analysis on antimicrobial resistance and relation with broad-spectrum rates were added in April 2019 after the first data analysis.

Following reviewer comments, additional analysis were added to the paper: appropriate indication of antibiotic prescription, appropriateness of duration of antibiotic prescription, and in patients with infections of the respiratory tract and urinary tract, guideline concordance of antibiotic prescriptions.

## SAP version 28 May 2018

## Background

Febrile illness is the most frequent reason for visiting the emergency department in children. The majority of children have a self-limiting disease and do not need treatment. The prevalence of serious bacterial infections ranges from 5 – 30% at the ED. Current guidelines state that there is no indication for oral antibiotics in children with fever without a clear diagnosis of bacterial infection. Nevertheless, antibiotic prescription rates in febrile children remain high and variation exists between countries.

## Objectives

- To study diversity in antibiotic prescription in febrile children visiting the ED

## Methods: study design

**Study design:**
Prospective observational study
This study is a prospectively planned analysis in the MOFICHE study (Management and Outcome of Febrile Illness in Children). MOFICHE is a prospective observational study using routine data. The need for informed consent was waived.

**Setting:**
12 Emergency Departments (EDs) in 8 countries

**Population:**
Children 0-18 years with fever (temperature >38.0 C) measured at ED or history of fever (<72 hours) before ED visit.

**Inclusion period:**
1 January 2017 – 1 April 2018, at least 12 months per study site.

**Sample size:**
According estimated patients visits, MOFICHE is expected to include 50,000 children. Pilot data estimated an antibiotic prescription rate of 30%. Using the rule of thumb of 10 events per variable, this study is large enough to study 120 determinants.

**Primary outcomes:**

- Antibiotic prescription at the ED or <24 hours after hospital admission
  - Defined as prescription of at least 1 systemic antibiotic (topical antibiotics are excluded)

**Secondary outcomes:**

- Broad-spectrum antibiotics vs narrow-spectrum antibiotics
- Intravenous/intramuscular vs oral route of administration

**Subgroup analysis:**

- Specific infections:
  - Lower respiratory tract infections
  - Upper respiratory tract: otitis media
  - Upper respiratory tract: tonsillitis / pharyngitis
  - Upper respiratory tract: other
- Final diagnosis groups:
  - Probable bacterial
  - Probable viral
  - Unknown bacterial/viral
- Patients with comorbidity (>=1) vs no comorbidity

## Methods: definitions and categorizations

- ***Definition broad-spectrum and narrow-spectrum***
  Antibiotic types will be classified using the anatomical therapeutic chemical classification (ATC). Since no clear, uniform definition of broad-spectrum and narrow-spectrum antibiotics exist, we have searched literature regarding antibiotic use in children. Second, we used an expert opinion including specialists in pediatric infectious diseases and general pediatricians (PERFORM consortium) to establish a classification in broad-spectrum and narrow-spectrum antibiotics (Table 1). Antibiotics to which resistance may be harmful were classified as broad-spectrum antibiotics.

- ***Final diagnosis (according to flowchart) (figure 1):***
  The final diagnosis is assigned using the flowchart, adapted to Herberg et al. 2016 [1]
  *Definite bacterial:* clinical bacterial symptoms and culture from sterile site with pathogenic bacteria
  *Probable bacterial:* clinical bacterial symptoms, no pathogenic bacteria identified from sterile site and CRP >60 mg/L.
  *Bacterial syndrome:* clinical bacterial symptoms, CRP ≤60 mg/L or no CRP performed
  *Definite viral:* clinical viral symptoms, identified virus that matches clinical symptoms and CRP ≤60mg/L

*Probable viral:* clinical viral symptoms, no identified virus and CRP ≤60 mg/L
*Viral syndrome:* clinical viral symptoms, CRP >60 mg/L or no CRP performed
*Unknown bacterial/viral:* children with symptoms that can be either bacteria/viral or with unclear diagnostic results
Co-infections of bacterial and viral infections were classified as bacterial.
*Trivial:* short duration of fever, no symptoms that can lead to another diagnosis and no diagnostics.
*Other:* other infection (e.g. malaria, fungal etc.)
*Other unclear:* unclear, does not match criteria for infection or inflammatory.
*Inflammatory syndrome:* matches criteria for inflammatory syndrome.

Final diagnosis was categorized in the following groups:

- (probable) bacterial: definite bacterial / probable bacterial / bacterial syndrome
- unknown bacterial/viral: unknown bacterial/viral
- (probable) viral: definite viral / probable viral / viral syndrome
- other: other infection / trivial / infection or inflammatory / inflammatory

- ***Categorization of infection focus:***
  - Upper respiratory infection: otitis media / tonsillitis/pharyngitis / other
  - Lower respiratory infection
  - Undifferentiated fever
  - Gastro-intestinal and surgical abdomen
  - Urinary tract
  - Sepsis / central nervous system
  - Soft tissue / musculoskeletal
  - Childhood exanthemas / flu-like illness
  - Other

- ***Triage urgency:***
  11 EDs use Manchester Triage System, 1 ED uses a local triage system (RUMC)
  - High urgency (MTS=immediate / very urgent / urgent, RUMC=U0, U1, U2, U3)
  - Low urgency (MTS=non-urgent /standard, RUMC=U4, U5)

- ***Comorbidity***
  - Chronic underlying condition that is expected to last at least 1 year[2]

- ***NICE alarming signs for identifying risk of serious illness*** [3]:
  - Presence of 0 alarming signs, 1 alarming sign or 2 or more alarming signs. Red alarming sings include:
    - Ill appearance: ill, moderately ill, irritable or uncomfortable
    - Moderate or severe work of breathing
    - Respiratory rate >60 /min
    - Age <3 months

        - Dehydration: dry mucous membranes, sunken eyes, reduced skin turgor or no tears
        - Non-blanching rash: petechiae or other non-blanching rash
        - Status epilepticus: seizures for >=30 minutes
        - Meningeal signs: presence of Kernig, Burdzinski, tripod phenomenon, neck stiffness, bulging fontanelle
        - Focal neurological signs
        - Abnormal consciousness: responsive to verbal stimulation, responsive to pain or unresponsive

- ***CRP level***
    - CRP is categorized as not performed / <20 / 20-60 / >60 mg/L.
      These cut-offs are chosen according to literature and to match with the flowchart for classification of the final diagnosis.[1,4]
- ***Chest X-ray:***
    - Abnormal chest X-ray:  focal infiltrate or consolidation / pleural effusion / diffuse abnormalities  /other
- ***Urinalysis:***
    - Abnormal urinalysis: leukocyturia or nitrite positive
- ***Antibiotic prescription mode:***
    - Antibiotic prescription mode: intravenous & intramuscular / oral
- ***Previous antibiotic use***
    - Use of therapeutic antibiotic use in 7 days before ED visit

## Methods: missing data

For this analysis, we will exclude patients with missing antibiotic prescription, missing focus of infection and missing final diagnosis.

We will explored missing data patterns and missingness for all variables for each ED. We will use multiple imputation by chained equations using the MICE package in R to impute all missing covariates. We will assume the variables to be 'missing at random' where missingness can be explained by other variables in the data. We will incorporate hospital, all covariates, outcome measures and other auxiliary variables in the imputation model.

Variables in the multiple imputation model:

| General characteristics | Markers of disease severity | Vital signs | Diagnostics | Treatment | Outcomes |
|---|---|---|---|---|---|
| Hospital | Triage urgency | Heart rate | CRP categories | Immediate life-saving interventions | Disposition |
| Age | Fever duration | Respiratory rate | Chest X-ray categories | Oxygen treatment | Final diagnosis |
| Sex | Capillary refill time | Temperature | Urinalysis categories | Inhalation medication | Focus of infection |
| Referral type (self / GP / emergency services / other) | Ill appearance | Oxygen saturation | Blood culture performed | Antibiotic prescription type | |
| Previous medical care (yes, primary care / yes, this ED / yes other secondary care) | Work of breathing | | Cerebrospinal fluid performed | Antibiotic prescription mode | |
| Season | Meningeal signs | | | Previous antibiotic treatment | |
| Arrival hours (morning / evening / night) | Focal neurology | | | | |
| Comorbidity | Non-blanching rash | | | | |
| | Dehydration | | | | |
| | Seizures | | | | |

# Methods: analysis

## Descriptive analysis

Description of study population including antibiotic prescription rate, broad-spectrum rate per ED. Antibiotic prescription rates per focus of infections and final diagnosis.

We will use frequencies (ranges between EDs), mean and standard deviation for normally distributed data, median and interquartile range for not normally distributed data.

Statistical differences will be assess using the Chi-squared test (categorical data), Mann-Whitney U test (ordinal data and non-normally distributed data), unpaired T-test (normally distributed data).

Data visualization for variation in antibiotic prescription rates according to different infections (lower and upper respiratory tract infections) and final diagnosis (probable bacterial, probable viral, unknown bacterial/viral).

## Multilevel logistic regression analysis

We will use multilevel logistic regression analysis with dichotomous outcome variable antibiotic prescription (yes/no) with setting level and patient level. We will included covariates on setting level and patient level.

### Setting level factors

As the number of EDs is limited (n=12) the number of setting variables is restricted (maximum of two variables). The following setting variables are available:

- Hospital type: teaching / university
- Supervising specialist: paediatrician / supervising specialist other than paediatrician
- Paediatric focused ED: yes/ no
- EDs with >50% self-referrals: yes / no
- Supervision: Patient discussed with supervisor during out of office hours: always / often / sometimes / never.
- Availability of primary care during out-of-office hours: yes / no
- Availability of point-of-care test of C-reactive protein (CRP): yes / no
- Availability of point-of-care rapid strep test: yes / no
- ED volume: Low volume <10,000 annual visits / intermediate volume 10,000 – 30,000 visits / high volume >30,000 annual visits

First, we have selected variables that varied between EDs and are related to antibiotic prescribing:

- ED volume, supervision, availability of point-of-care tests (rapid strep test and CRP) and availability of primary care during out-of-office hours.

We will include hospital-level factors if they improve model  0 in a univariate analysis (Likelihood ratio test, P<0.05).

## Patient level factors

We will include the following patient-level variables. These variables are a-priori selected and are either related with case-mix, disease severity or antibiotic prescribing.

**General characteristics:**

- Age (continuous)
- Gender (male / female)
- Referral (referred / self-referred)
- Season (spring / summer / autumn / winter)
- Comorbidity (yes / no)

**Markers for disease severity:**

- Triage urgency (high / low)
- Fever duration in days (continuous)
- Presence of NICE guideline red warning signs (0 /1 / >=2)

**Diagnostics:**

- CRP (not performed / <20 / 20-60 / >60 mg/L)
- Chest x-ray (not performed / normal / abnormal)
- Urinalysis (not performed / normal / abnormal)

**Cause and focus of infection:**

- Focus of infection (upper respiratory tract / lower respiratory tract / gastro-intestinal tract / urinary tract / undifferentiated fever / skin & musculoskeletal / sepsis & central nervous system / flu like illness & childhood exanthemas / inflammatory & other).
- Cause of infection (presumed bacterial / unknown bacterial viral / presumed viral / other)

For the outcome broad-spectrum vs narrow-spectrum antibiotics we will also include previous antibiotic use as covariate.

# Model building

## Data preparation

We will evaluate linearity of continuous variables (age, duration of fever) using restricted cubic splines.

## Model 0 'empty model'

Model only including random intercept for ED.

## Model 1

Model 0 + selected hospital-level predictors.

Hospital-level predictors are tested in a univariate analysis whether they are significant (Likelihood ratio test, p<0.05) and we will select two covariates on the setting level that are significant.

### Model 2

Add all patient-level predictors to model 1.

## Measures of variation

- Standardized antibiotic prescription rates
  Ratio between observed antibiotic prescriptions (based on model 0, only including random intercept for ED) and expected antibiotic prescriptions (based on model 2).

  Standardized rates >1 demonstrate higher antibiotic prescription rates than expected according to the model, and standardized rates <1 indicate lower rates of antibiotic prescription than expected according to the model.

- Median Odds Ratios (MOR) [5]
  - The MOR is a measure of variation between clusters
  - The more reflects the difference in prescription of antibiotics comparing similar patients attending EDs with high prescribing vs EDs with low prescribing

$$MOR = \exp\left[\sqrt{2 \times \tau^2} \times 0.6745\right]$$

$\tau^2$ is the hospital-level variance.

If MOR is equal to 1, there is no variation between hospitals. If there is considerable between-hospital variation, the MOR will be large.

# Table 1: Definition of broad and narrow antibiotics (MOFICHE)

**Table 1: Definition of broad and narrow antibiotics (MOFICHE)** [6-9]

| Narrow | | | Broad | |
|---|---|---|---|---|
| ***Beta-lactamase sensitive penicillins*** | benzylpenicillin (pen G), pheneticillin, benzathine, phenoxymethylpenicillin, benzathine benzylpenicillin, phenoxymethylpenicillin (pen V) | | ***Combinations of penicillins, including beta-lactamase inhibitors*** | ampicillin and beta-lactamase inhibitor, amoxicillin and beta-lactamase inhibitor, piperacillin and beta-lactamase inhibitor |
| ***Beta-lactamase resistant penicillins*** | cloxacillin, flucloxacillin, oxacillin | | ***2nd- generation cephalosporins*** | cefaclor, cefprozil, cefuroxime |
| ***Penicillins with extended spectrum*** | amoxicillin, ampicillin, piperacillin | | ***3rd –generation cephalosporins*** | cefotaxime, ceftazidime, cefixime, ceftriaxone, cefdinir, cefpodoxime |
| ***1st -generation cephalosporins*** | cefazolin, cefadroxil, cefalexin, | | ***Macrolides*** | erythromycin, azithromycin, clarithromycin, josamycin, midecamycin |
| ***Sulfonamides/ trimethoprim*** | trimethoprim | | ***Sulfonamides/ trimethoprim*** | sulfamethoxazole-trimethoprim |
| ***Nitrofurans*** | furazolidone, nitrofurantoin | | ***Tetracyclines*** | doxycycline, tetracycline |
| ***Other*** | colistin (polymxin), tazobactam | | ***Lincosamides*** | clindamycin |
| | | | ***Carbapenems*** | meropenem |
| | | | ***Quinolones*** | ciprofloxacin, levofloxacin, ofloxacin |
| | | | ***Aminoglycosides*** | gentamicin, tobramycin, amikacin, neomycin |
| | | | ***Glycopeptides*** | vancomycin, teicoplanin |
| | | | ***Imidazole derivates*** | metronidazole |
| | | | ***Other*** | rifampicin |

## Figure 1: categorization of final diagnosis

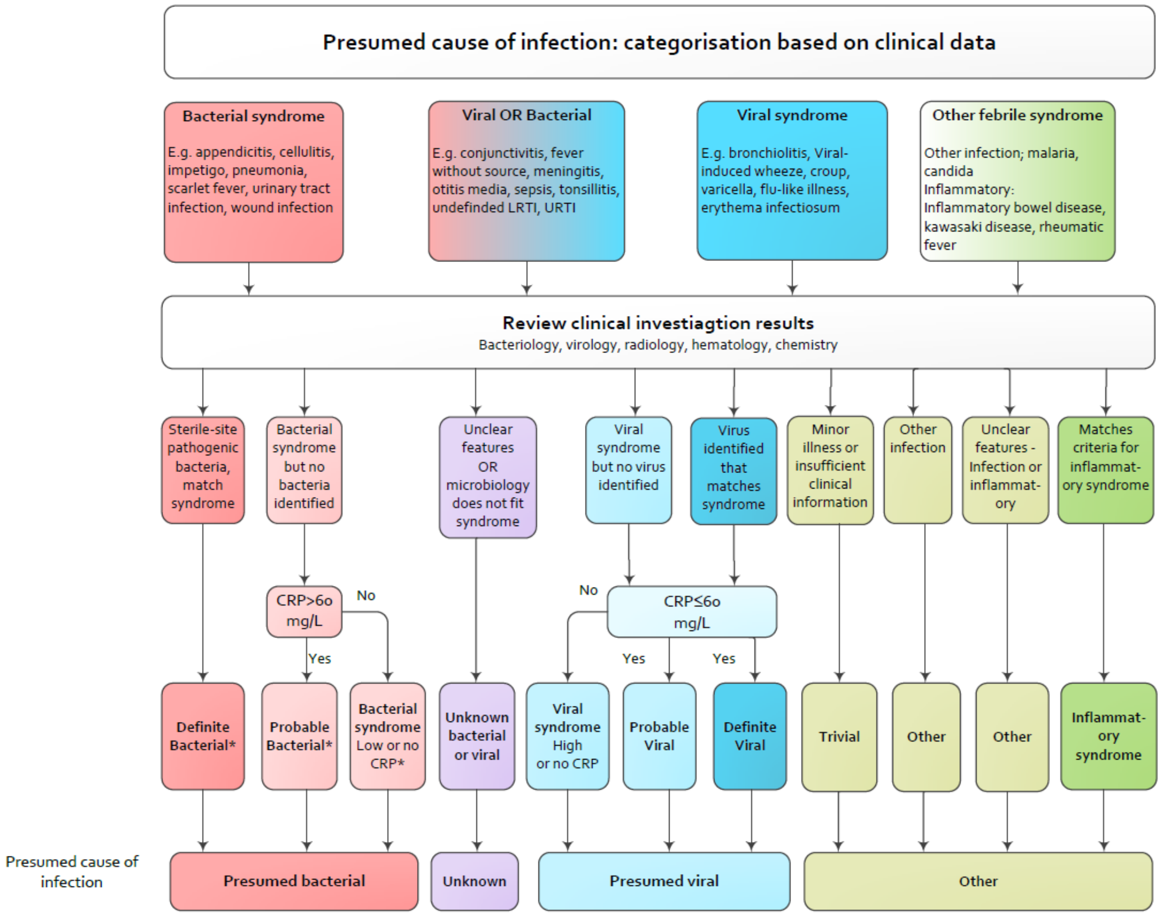


*CRP, C-reactive protein; LRTI, lower respiratory tract infection; URTI, upper respiratory tract infection*

*Patients could have identified viral co-infection

The diagnosis definite bacterial infection was assigned only when a sterile site culture identified pathogenic bacteria. The diagnosis ‘probable bacterial infection’ was allocated when a bacterial syndrome was suspected but no bacteria was identified and CRP was above 60 mg/L. Patients with clinical bacterial symptoms and CRP ≤60 mg/L or no CRP were listed as ‘bacterial syndrome’. Children with suspected viral infections were classified as ‘viral syndrome’ (no CRP or CRP >60 mg/L) or ‘definite viral’ (CRP ≤60 mg/L) when a virus was identified that matched the clinical symptoms. Children with a viral syndrome, but no identified virus were classified as ‘probable viral’ (CRP ≤60 mg/L). Children who did not fit the definitions were classified as unknown bacterial/viral. Children with mixed infections (co-infection of bacterial and viral) were classified as bacterial.

# References

1. Herberg JA, Kaforou M, Wright VJ, et al. Diagnostic Test Accuracy of a 2-Transcript Host RNA Signature for Discriminating Bacterial vs Viral Infection in Febrile Children. *Jama* 2016; **316**(8): 835-45.
2. Simon TD, Cawthon ML, Stanford S, et al. Pediatric medical complexity algorithm: a new method to stratify children by medical complexity. *Pediatrics* 2014; **133**(6): e1647-54.
3. The National Institute for Health and Care Excellence. Fever in under 5s: assessment and initial managment CG160 May 2013. August 2017 2013. https://www.nice.org.uk/guidance/cg160.
4. Van den Bruel A, Thompson MJ, Haj-Hassan T, et al. Diagnostic value of laboratory tests in identifying serious infections in febrile children: systematic review. *Bmj* 2011; **342**: d3082.
5. Sanagou M, Wolfe R, Forbes A, Reid CM. Hospital-level associations with 30-day patient mortality after cardiac surgery: a tutorial on the application and interpretation of marginal and multilevel logistic regression. *BMC Med Res Methodol* 2012; **12**: 28.
6. Aabenhus R, Siersma V, Hansen MP, Bjerrum L. Antibiotic prescribing in Danish general practice 2004-13. *J Antimicrob Chemother* 2016; **71**(8): 2286-94.
7. Sarpong EM, Miller GE. Narrow- and Broad-Spectrum Antibiotic Use among U.S. Children. *Health Serv Res* 2015; **50**(3): 830-46.
8. Hersh AL, Shapiro DJ, Pavia AT, Shah SS. Antibiotic prescribing in ambulatory pediatrics in the United States. *Pediatrics* 2011; **128**(6): 1053-61.
9. Gerber JS, Ross RK, Bryan M, et al. Association of Broad- vs Narrow-Spectrum Antibiotics With Treatment Failure, Adverse Events, and Quality of Life in Children With Acute Respiratory Tract Infections. *Jama* 2017; **318**(23): 2325-36.
