# Supplementary material for: Variation in antibiotic prescription rates in febrile children presenting to emergency departments across Europe (MOFICHE): A multicentre observational study
Source: PLoS Med. 2020 Aug 19;17(8):e1003208. doi: 10.1371/journal.pmed.1003208 (PMC7444592; doi:10.1371/journal.pmed.1003208)
Supplement: S3 Text — (PDF) [file pmed.1003208.s006.pdf]

Supplemental 3: Ethics committees of participating hospitals

Ethical approval:

The study was approved by all the participating hospitals. No informed consent was needed for this study.

Austria (Ethikkommission Medizinische Universitat Graz, ID: 28-518 ex 15/16),

Germany (Ethikkommission Bei Der LMU München, ID: 699-16),

Greece (Ethics committee, ID: 9683/18.07.2016),

Latvia (Centrala medicinas etikas komiteja, ID: 14.07.201 6. No. Il 16-07 -14),

Slovenia (Republic of Slovenia National Medical Ethics Committee, ID: ID: 0120-483/2016-3),

Spain (Comité Autonómico de Ética de la Investigación de Galicia, ID: 2016/331),

The Netherlands (Commissie Mensgebonden onderzoek, ID: NL58103.091.16),

United Kingdom (Ethics Committee, ID: 16/LO/1684, IRAS application no. 209035, Confidentiality advisory group reference: 16/CAG/0136).
In United Kingdom an "opt-out" procedure was used for this study.
