# Supplementary material for: Variation in antibiotic prescription rates in febrile children presenting to emergency departments across Europe (MOFICHE): A multicentre observational study
Source: PLoS Med. 2020 Aug 19;17(8):e1003208. doi: 10.1371/journal.pmed.1003208 (PMC7444592; doi:10.1371/journal.pmed.1003208)
Supplement: S4 Text — (PDF) [file pmed.1003208.s007.pdf]

Supplemental 4 - Hospital characteristic

Hospital	Country, city	Hospital type	PICU available in hospital	Total annual paediatric ED visits	Total period of inclusion	Period of inclusion per month	Number of included patients	Availability of primary care out-of-office hours	Availability of C-reactive protein POCT	Availability of Group A streptococcal POCT	Supervision: patient discussed during out-of-office hours
Medizinische Universität Graz	Austria, Graz	University	Yes	10,000-30,000	1-1-2017 – 31-12-2018	10 days	2109	No	Yes	Yes	Sometimes
Dr. von Hauner Children's Hospital	Germany, Munich	Teaching	Yes	10,000-30,000	1-1-2017 – 31-12-2018	1 week	1124	Yes	Yes	No	Sometimes
P. and A. Kyriakou Children’s Hospital	Greece, Athens	University	Yes	>30,000	1-1-2017 – 1-5-2018	1-2 weeks	4334	No	No	Yes	Rarely/never
Children clinical university hospital	Latvia, Riga	Teaching	Yes	>30,000	1-1-2017 – 31-12-2018	All	8486	No	Yes	Yes	Rarely/never
Univerzitetni Klinični Center	Slovenia, Ljubljana	University	Yes	<10,000	1-1-2017 – 31-12-2018	All	3593	Yes	Yes	Yes	Often
Hospital Clínico Universitario	Spain, Santiago de Compostela	University	Yes	>30,000	1 Jan 2017 – 1 May 2018	1-2 weeks	3301	Yes	Yes	Yes	Often
Erasmus MC-Sophia Children’s Hospital	The Netherlands, Rotterdam (NL, 2)	University	Yes	<10,000	1 Jan 2017 – 1 Apr 2018	All	1468	Yes	Yes	No	Always
RadboudUMC	The Netherlands, Nijmegen (NL, 1)	University	Yes	<10,000	1 Jan 2017 – 1 Apr 2018	All	634	Yes	No	No	Always
Canisius Wilhelmina Hospital	The Netherlands, Nijmegen (NL, 3)	Teaching	No	<10,000	1-1-2017 – 31-12-2018	2 weeks	401	Yes	No	No	Always
Alder Hey Children’s Hospital	United Kingdom, Liverpool (UK, 1)	Teaching	Yes	>30,000	1-1-2017 – 31-12-2018	1 week	1423	Yes	No	No	Sometimes
St. Mary’s Hospital	United Kingdom, London (UK, 3)	University	Yes	10,000-30,000	1-1-2017 – 31-12-2018	All	5206	Yes	No	No	Often
Great North Children’s Hospital	United Kingdom, Newcastle upon Tyne (UK, 2)	University	Yes	>30,000	1-4-2017 – 1-4-2018	2 weeks	3571	Yes	No	No	Often

ED, emergency department; PICU, paediatric intensive care unit; POCT, point-of-care test
