# Supplementary material for: Variation in antibiotic prescription rates in febrile children presenting to emergency departments across Europe (MOFICHE): A multicentre observational study
Source: PLoS Med. 2020 Aug 19;17(8):e1003208. doi: 10.1371/journal.pmed.1003208 (PMC7444592; doi:10.1371/journal.pmed.1003208)
Supplement: S5 Text — (PDF) [file pmed.1003208.s008.pdf]

Supplemental file 5 - Broad-spectrum and narrow-spectrum definition

Definition of broad-spectrum and narrow-spectrum antibiotics (MOFICHE)[1-6]

Narrow		Broad	
Beta-lactamase sensitive penicillins	benzylpenicillin (pen G), pheneticillin, benzathine, phenoxymethylpenicillin, benzathine benzylpenicillin, phenoxymethylpenicillin (pen V)	***Combinations of penicillins, including beta-lactamase inhibitors***	ampicillin and beta-lactamase inhibitor, amoxicillin and beta-lactamase inhibitor, piperacillin and beta-lactamase inhibitor
Beta-lactamase resistant penicillins	cloxacillin, flucloxacillin, oxacillin	***2nd- generation cephalosporins***	cefaclor, cefprozil, cefuroxime
Penicillins with extended spectrum	amoxicillin, ampicillin, piperacillin	***3rd –generation cephalosporins***	cefotaxime, ceftazidime, cefixime, ceftriaxone, cefdinir, cefpodoxime
1st -generation cephalosporins	cefazolin, cefadroxil, cefalexin,	***Macrolides***	erythromycin, azithromycin, clarithromycin, josamycin, midecamycin
Sulfonamides/ trimethoprim	trimethoprim	***Sulfonamides/ trimethoprim***	sulfamethoxazole-trimethoprim
Nitrofurans	furazolidone, nitrofurantoin	***Tetracyclines***	doxycycline, tetracycline
Other	colistin (polymxin), tazobactam	***Lincosamides***	clindamycin
		Carbapenems	meropenem
		Quinolones	ciprofloxacin, levofloxacin, ofloxacin
		Aminoglycosides	gentamicin, tobramycin, amikacin, neomycin
		Glycopeptides	vancomycin, teicoplanin
		Imidazole derivates	metronidazole
		Other	rifampicin

1. Aabenhus R, Siersma V, Hansen MP, Bjerrum L. Antibiotic prescribing in Danish general practice 2004-13. J Antimicrob Chemother. 2016;71(8):2286-94. PubMed PMID: 27107098.
2. Sarpong EM, Miller GE. Narrow- and Broad-Spectrum Antibiotic Use among U.S. Children. Health Serv Res. 2015;50(3):830-46. PubMed PMID: 25424240.
3. Hersh AL, Shapiro DJ, Pavia AT, Shah SS. Antibiotic prescribing in ambulatory pediatrics in the United States. Pediatrics. 2011;128(6):1053-61. PubMed PMID: 22065263.
4. European Centre for Disease Prevention and Control. Surveillance of antimicrobial resistance in Europe - Annual report of the European Antimicrobial Resistance Surveillance Network (EARS-Net): European Centre for Disease Prevention and Control; 2018 [cited 2019 15-03-2019]. Available from: https://ecdc.europa.eu/sites/portal/files/documents/EARS-Net-report-2017-update-jan-2019.pdf.
5. Gerber JS, Ross RK, Bryan M, Localio AR, Szymczak JE, Wasserman R, et al. Association of Broad- vs Narrow-Spectrum Antibiotics With Treatment Failure, Adverse Events, and Quality of Life in Children With Acute Respiratory Tract Infections. JAMA. 2017;318(23):2325-36. PubMed PMID: 29260224.
6. van de Maat J, van de Voort E, Mintegi S, Gervaix A, Nieboer D, Moll H, et al. Antibiotic prescription for febrile children in European emergency departments: a cross-sectional, observational study. Lancet Infect Dis. 2019. PubMed PMID: 30827808.
