# Supplementary material for: Variation in antibiotic prescription rates in febrile children presenting to emergency departments across Europe (MOFICHE): A multicentre observational study
Source: PLoS Med. 2020 Aug 19;17(8):e1003208. doi: 10.1371/journal.pmed.1003208 (PMC7444592; doi:10.1371/journal.pmed.1003208)
Supplement: S6 Text — (PDF) [file pmed.1003208.s009.pdf]

Supplemental file 6 – Local guidelines of antibiotic treatment

1st choice antibiotic for uncomplicated infections:

otitis media, lower respiratory tract infections, other respiratory infections:

All EDs (apart from UK, 3): 1st choice amoxicillin

UK, 3: 1st choice amoxicillin-clavulanate

1st choice antibiotic for uncomplicated infections:

tonsillitis/pharyngitis:

All EDs (apart from UK, 3): penicillin. In these EDs, amoxicillin was also defined as appropriate treatment for tonsillitis/pharyngitis.

UK, 3: <8 year: amoxicillin-clavulanate. >8 year: penicillin

Local recommendations for oral treatment of urinary tract infections

Infection	Recommended antibiotic type	ED
Urinary tract	Amoxicillin/clavulanic acid	Germany, Munich
		NL, Rotterdam
		NL, Nijmegen, Canisius
		NL, Nijmegen, Radboud
	Cefuroxime	Greece, Athens
		Latvia, Riga
		Spain, Santiago
	Cephalexine	UK, Liverpool
		UK, London
		UK, Newcastle
	Cephalexine OR Amoxicillin/clavulanic acid	Austria, Graz
	Suflonamides/trimethoprim OR nitrofurantoin	Slovenia, Ljubljana
