# Supplementary material for: Variation in antibiotic prescription rates in febrile children presenting to emergency departments across Europe (MOFICHE): A multicentre observational study
Source: PLoS Med. 2020 Aug 19;17(8):e1003208. doi: 10.1371/journal.pmed.1003208 (PMC7444592; doi:10.1371/journal.pmed.1003208)
Supplement: S7 Text — (PDF) [file pmed.1003208.s010.pdf]

Supplemental file 7 – Details of the adjusted model

Details of the adjusted model:

Outcome: antibiotic prescription rate
Null model: glmer (antibiotics_yn ~ (1|hospital)

Adjusted model: glmer (antibiotics_yn ~ (1|hospital) + ns(age,3) + sex + referral +

comorbidity + season + triage urgency 2cats + fever duration + temp>=38 + alarmings sings + CRP categories + chest X-ray categories + urinalysis categories + focus of infection + cause of infection, family=binomial)

Outcome: broad-spectrum rate vs narrow-spectrum
Null model: glmer (ab_broad ~ (1|hospital)

Adjusted model: glmer (ab_broad ~(1|hospital) + ns(ag,3) + sex + referral +

comorbidity + season + triage urgency + fever duration + temp>=38 + alarming signs + CRP categories + chest X-ray categories + urinalysis categories + focus of infection + cause of infection + previous antibiotic treatment, family=binomial)

Outcome: intravenous/intramuscular vs oral mode of prescription
Null model: glmer (ab_mode ~ (1|hospital)

Adjusted model: glmer (ab_mode ~ (1|hospital) + ns(age,3) + sex + referral +

comorbidity + season + triage urgency + fever duration + temp>=38 + alarming signs + CRP categories + chest X-ray categories + urinalysis categories + focus of infection + cause of infection, family=binomial)
