# Supplementary material for: Variation in antibiotic prescription rates in febrile children presenting to emergency departments across Europe (MOFICHE): A multicentre observational study
Source: PLoS Med. 2020 Aug 19;17(8):e1003208. doi: 10.1371/journal.pmed.1003208 (PMC7444592; doi:10.1371/journal.pmed.1003208)
Supplement: S8 Text — (PDF) [file pmed.1003208.s011.pdf]

Supplemental file 8 - National and hospital antimicrobial resistance data

Methods:

Since antimicrobial resistance patterns vary greatly between European countries, standardized rates of broad-spectrum vs narrow-spectrum antibiotics were compared with antimicrobial resistance data of invasive isolates for specific pathogens on a national level and the local hospital [1, 2]: staphylococcus aureus (methicillin resistance) for all patients, Streptococcus pneumoniae (resistance for penicillins and macrolides) for patients with lower respiratory tract infections and Escherichia coli (combined resistance for fluoroquinolones, 3rd-generation cephalosporins and aminoglycosides) for patients with urinary tract infections. Since antimicrobial resistance patterns could vary between hospitals in the same country, we also compared high broad-spectrum prescriptions with data of the local methicillin resistance for invasive isolates of S. aureus. For hospital resistance data, we focused on methicillin resistance for S. aureus as these data were uniformly collected. Correlations were calculated using the two-tailed Spearman's Rank coefficient (ρ). A p-value below 0.05 was determined as significant.

Results:

Higher standardized rates for broad-spectrum antibiotics were not related to higher antimicrobial resistance percentages on a national level (resistance S. aureus (ρ -0.07 95% CI (-0.62 to 0.53), p=0.83), S. pneumoniae (ρ -0.31 (95% CI -0.77 to 0.35), p=0.35) and E. coli (ρ 0.07 95%CI (-0.52 to 0.62), p=0.82) or on a hospital level (methicillin resistance rates for S. aureus ρ 0.12 (95% CI -0.48 to 0.65), p=0.70).

National antimicrobial resistance data extracted from ECDC 2017[1]

N: number of isolates tested

S. aureus - resistance to methicillin

**Country**	**N**	**Resistance %**	**95% CI**
Austria	3158	5.9	(5-7)
Germany	12021	9.1	(9-10)
Greece	822	38.4	(35-42)
Latvia	210	5.7	(3-10)
Netherlands	2694	1.5	(1-2)
Slovenia	576	9	(7-12)
Spain	1804	25.3	(23-27)
United Kingdom	8883	6.9	(6-7)

S. Pneumoniae - resistance to penicillins and macrolides

**Country**	**N**	**Resistance %**	**95% CI**
Austria	457	3.3	(2-5)
Germany	1803	2.4	(2-3)
Greece*	-	-	-
Latvia	28	3.6	(0-18)
Netherlands	1297	1.1	(1-2)
Slovenia	216	6.5	(4-11)
Spain	676	12.4	(10-15)
United Kingdom	3885	2	(2-2)

*Data from Greece not available

E.coli - combined resistance to fluoroquinolones, 3rd generation cephalosporins and aminoglycosides

**Country**	**N**	**Resistance %**	**95% CI**
Austria	5071	3.3	(3-4)
Germany	20610	3.7	(3-4)
Greece	1463	9.8	(8-11)
Latvia	197	11.2	(7-16)
Netherlands	6681	1.9	(2-2)
Slovenia	1381	6.3	(5-8)
Spain	5551	5.5	(5-6)
United Kingdom	26808	4.1	(4-4)

Hospital antimicrobial resistance rates for S. aureus - resistance to methicillin for invasive isolates

**Country**	**N**	**N Resistant isolates**	**Resistance %**	**Population**
Austria	237	22	9%	Children & adults
Germany	13	0	0%	<18 y
Greece	23	5	22%	<18 y
Latvia	5	1	20%	<18 y
Slovenia	16	0	0%	<18 y
Spain	11	0	0%	<18 y
NL, 1 and NL, 3	55	1	2%	Children & adults
NL, 2	37	2	5%	<18 y
UK, 1	50	10	20%	<18 y
UK, 2	61	3	5%	<16 y
UK, 3	68	5	7%	<16 y

NL, the Netherlands; UK, United Kingdom, y, year

References

1. European Centre for Disease Prevention and Control. Surveillance of antimicrobial resistance in Europe - Annual report of the European Antimicrobial Resistance Surveillance Network (EARS-Net): European Centre for Disease Prevention and Control; 2018 [cited 2019 15-03-2019]. Available from: https://ecdc.europa.eu/sites/portal/files/documents/EARS-Net-report-2017-update-jan-2019.pdf.
2. World Health Organization. Prioritization of pathogens to guide discovery, research and development of new antibiotics for drug-resistant bacterial infections, including tuberculosis. : World Health Organization; 2017 [cited 2019 25-03-2019]. Available from: https://www.who.int/medicines/areas/rational_use/PPLreport_2017_09_19.pdf?ua=1.
