# Supplementary material for: Variation in antibiotic prescription rates in febrile children presenting to emergency departments across Europe (MOFICHE): A multicentre observational study
Source: PLoS Med. 2020 Aug 19;17(8):e1003208. doi: 10.1371/journal.pmed.1003208 (PMC7444592; doi:10.1371/journal.pmed.1003208)
Supplement: S9 Text — (PDF) [file pmed.1003208.s012.pdf]

Supplemental file 9 – Descriptive characteristics of cases with complete outcomes and cases with missing outcomes

	Complete cases for antibiotics, infection focus, final diagnosis (n=37742)		Cases with missing antibiotics, infection focus or final diagnosis (n=738)		
	N (%)	N missing	N (%)	N missing	p-value
**Age in years,** median (IQR)	2.8 (1.3 - 5.5)		3.1 (1.5 - 5.9)		0.06*
**Male**	20700 (54.8)	1	410 (55.6)		0.73
**Comorbidity**	6377 (16.9)	361	117 (15.9)	9	0.5
**Season**					<0.05
Winter	13424 (35.6)		378 (51.2)		
Spring	9546 (25.3)		115 (15.6)		
Summer	6089 (16.1)		104 (14.1)		
Autumn	8683 (23.0)		141 (19.1)		
**Triage urgency**		1172		4	<0.05
High: intermediate, very urgent, immediate,	13074 (34.6)		145 (19.6)		
Low: standard, non-urgent	23496 (62.3)		589 (79.8)		
**Referred**	15885 (42.1)	1152	191 (25.9)		<0.05
**Fever duration in days**, median (IQR)	0.5 (1.5 - 3)	2719	0.5 (1.5-3.0)	56	0.69*
**NICE "red traffic lights" (warning signs)**					
Ill appearance	5911 (15.7)	1677	96 (13.0)	44	0.08
Work of breathing	3231 (8.6)	4839	32 (4.3)	34	<0.05
Dehydration	1881 (4.9)	6916	20 (2.7)	61	<0.05
Rash: petechiae/non-blanching	1095 (2.9)	4352	17 (2.3)	61	0.32
Decreased consciousness	201 (0.5)	367	0 (0.0)	23	0.09
Meningeal signs	137 (0.4)	1993	0 (0.0)	51	0.19
Focal neurology	130 (0.3)	2404	3 (0.4)	0.4	1
Status epilepticus	64 (0.2)	1129	2 (0.3)	12	0.83
**C-reactive protein (CRP)**					<0.05
No CRP performed	20769 (55.0)		505 (68.4)		
<20 mg/L	9124 (24.2)		79 (10.7)		
20-60 mg/L	4459 (11.8)		73 (9.9)		
>60 mg/L	3390 (8.9)		81 (11.0)		
**Chest x-ray**					0.1
No	32433 (85.9)		618 (83.7)		
Normal	2059 (5.5)		53 (7.2)		
Abnormal	3250 (8.6)		67 (9.1)		
**Urinalysis**					<0.05
No	28360 (75.1)		622 (84.3)		
Normal	7548 (20.0)		96 (13.0)		
Abnormal	1834 (4.9)		20 (2.7)		
**Therapeutic antibiotics use in last 7 days***	4206 (11.1)	743	131 (17.8)	32	<0.05
**Antibiotic treatment duration, days, median (IQR)**	7 (5-10)	2163	9 (7-10)	3	<0.05*
**Antibiotics prescribed at ED visit or 1st day of hospital admission***	12239 (32.4)		59 (7.9)	484	<0.05
Narrow-spectrum	5738 (15.2)	85	34 (4.6)		0.14
Broad-spectrum	6416 (17.0)		25 (3.4)		
**Antibiotic administration***		197			<0.05
Oral	8195 (21.7)		54 (7.3)		

Intravenous/intramuscular	3847 (10.2)		5 (0.7)		
**Admission***	9707 (25.7)	26	186 (25.2)	30	0.78
**ICU admission***	154 (0.4)	26	4 (0.5)	39	0.73
**Focus of infection**				27	<0.05
Upper respiratory tract	19740 (52.3)		379 (51.4)		
Lower respiratory tract	5552 (14.7)		100 (13.6)		
Gastro-intestinal /surgical abdomen	3939 (10.4)		49 (6.6)		
Undifferentiated fever	2911 (7.7)		84 (11.4)		
Flu like illness/exanthemas	1863 (4.9)		25 (3.4)		
Urinary tract	1326 (3.5)		27 (3.7)		
Soft tissue/musculoskeletal	955 (2.5)		27 (3.7)		
Sepsis/central nervous system	291 (0.8)		0 (0.0)		
Inflammatory	145 (0.4)		1 (0.1)		
Other	1020 (2.7)		19 (2.6)		
**Final diagnosis**				272	<0.05
Probable viral	21441 (56.8)		45 (6.1)		
Definite bacterial	1566 (4.2)		10 (1.4)		
Probable bacterial	6899 (18.3)		64 (8.7)		
Unknown bacterial/viral	5533 (14.7)		331 (44.9)		
Other	2303 (6.1)		16 (2.2)		

*Unpaired t-test
