# Supplementary material for: Variation in antibiotic prescription rates in febrile children presenting to emergency departments across Europe (MOFICHE): A multicentre observational study
Source: PLoS Med. 2020 Aug 19;17(8):e1003208. doi: 10.1371/journal.pmed.1003208 (PMC7444592; doi:10.1371/journal.pmed.1003208)

Supplemental file 11 - Variation of antibiotic and broad-spectrum prescription in lower respiratory, otitis media, tonsillitis/pharyngitis and other upper respiratory tract infections

Results:

Antibiotic and broad-spectrum prescriptions varied for respiratory tract infections. In particular, antibiotic prescriptions varied for otitis media (80.7%, range EDs 39.8-92.5%) and tonsillitis/pharyngitis (51.3%, range EDs 14.8-82.3%) (Fig A). After adjustment of general characteristics, disease severity, CRP and cause of infection, variability remained in antibiotic prescription rates for patients with lower respiratory tract infection (range standardized rates 0.71-1.32, MOR 4.14), otitis media (range standardized rate: 0.73-1.78, MOR 4.48), tonsillitis/pharyngitis (range standardized rate: 0.63-1.85, MOR 3.54) and other upper respiratory tract infections (range standardized rate 0.39-2.77, MOR 3.14) (Fig B). These differences increased for broad-spectrum vs narrow-spectrum antibiotics in the majority of the different groups (range standardized rates: lower respiratory tract: 0.50-1.71, otitis media: 0.42-3.82, tonsillitis/pharyngitis 0.34-3.43, other upper respiratory 0.39-1.51) (Fig C).

Figure A: Range of antibiotic prescriptions (A) and broad−spectrum prescriptions (B) per emergency department (ED) for lower and upper respiratory tract infections

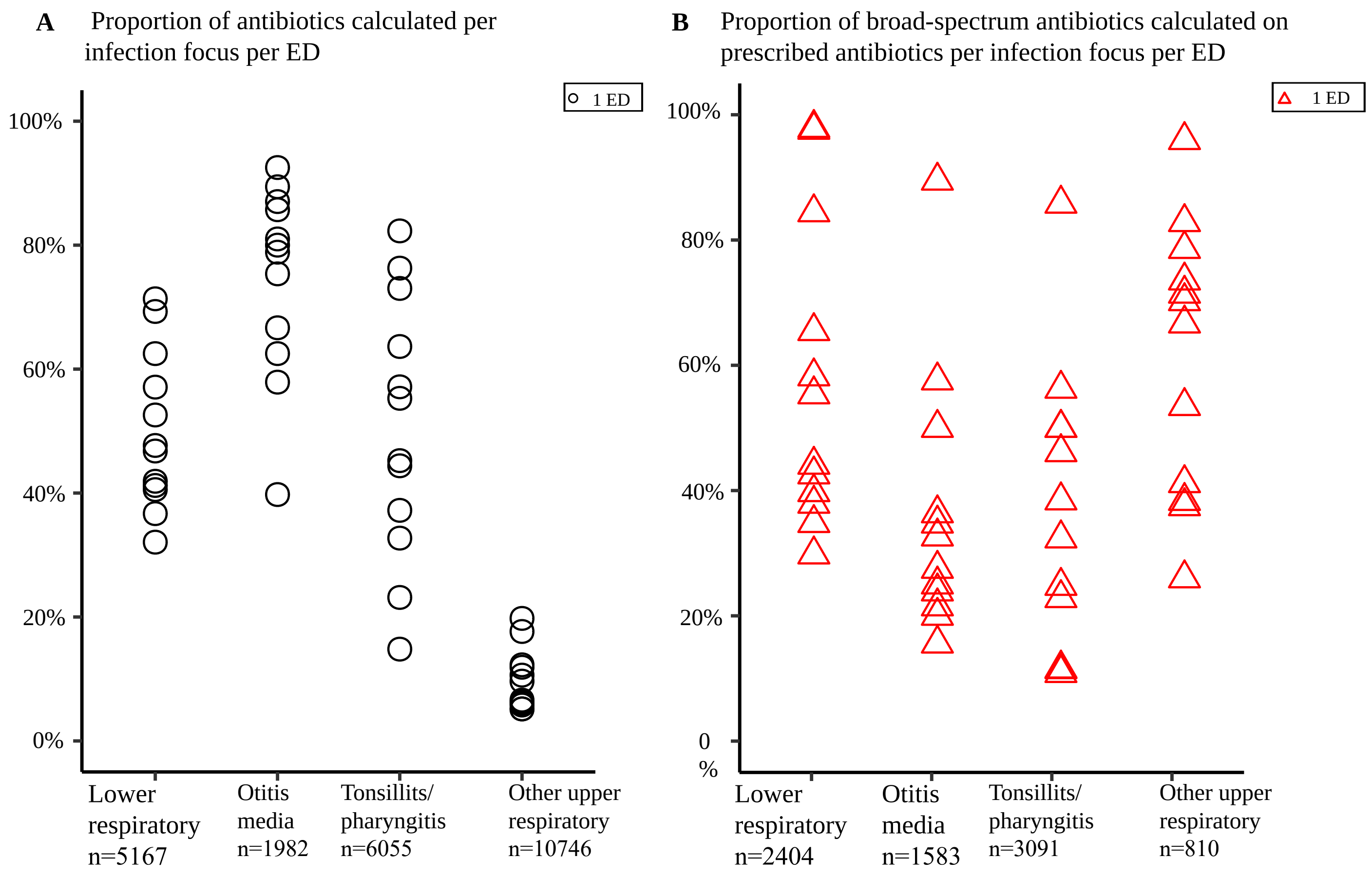

Figure B - Heat map of standardized antibiotic prescription rates stratified for lower respiratory, otitis media, tonsillitis/pharyngitis and other upper respiratory tract infections (95% CI)

All adjusted for age, sex, season, comorbidity, fever duration, warning sings, CRP, cause of infection.
*Also adjusted for chest X-ray and triage urgency

EDs	**Lower respiratory*** n=5167	**Otitis media** n=1982	**Tonsillitis/pharyngitis** n=6055	**Other upper respiratory** n=10746
UK, 2	1.32 (1.28-1.36)	1.45 (1.40-1.50)	1.85 (1.81-1.89)	1.50 (1.41-1.58)
UK, 3	1.31 (1.28-1.34)	1.78 (1.72-1.84)	1.12 (1.10-1.14)	2.77 (2.68-2.86)
UK, 1	1.24 (1.19-1.30)	1.10 (0.96-1.24)	1.33 (1.27-1.39)	1.55 (1.44-1.67)
NL, 2	1.20 (1.15-1.24)	0.73 (0.67-0.78)	1.21 (1.12-1.30)	1.34 (1.27-1.41)
NL, 1	1.14 (1.07-1.21)	1.01 (0.88-1.13)	1.05 (0.89-1.21)	1.55 (1.45-1.66)
Germany	1.07 (1.01-1.12)	0.81 (0.76-0.87)	0.93 (0.89-0.98)	1.00 (0.93-1.07)
Spain	1.03 (0.99-1.06)	0.99 (0.97-1.02)	0.84 (0.82-0.87)	1.26 (1.18-1.33)
Greece	1.02 (1.00-1.05)	1.71 (1.67-1.76)	1.08 (1.05-1.11)	1.30 (1.25-1.35)
NL, 3	0.99 (0.91-1.06)	0.95 (0.83-1.08)	0.69 (0.54-0.85)	0.92 (0.77-1.08)
Latvia	0.81 (0.79-0.82)	0.91 (0.89-0.93)	0.83 (0.82-0.84)	0.55 (0.53-0.57)
Slovenia	0.79 (0.77-0.81)	0.94 (0.90-0.99)	0.63 (0.60-0.65)	0.39 (0.36-0.42)
Austria	0.71 (0.67-0.74)	1.23 (1.17-1.29)	1.08 (1.05-1.10)	0.54 (0.50-0.58)

Scale

Min. 1.0 2.0 Max.

Figure C - Heat map of standardized broad-spectrum vs narrow-spectrum prescription rates stratified for lower respiratory, otitis media, tonsillitis/pharyngitis and other upper respiratory tract infections (95% CI)

All adjusted for age, sex, season, comorbidity, fever duration, warning sings, CRP, cause of infection and previous antibiotic use
*Also adjusted for chest X-ray

EDs	**Lower respiratory*** n=2404	**Otitis media** n=1583	**Tonsillitis/pharyngitis** n=3091	**Other upper respiratory** n=810
UK, 3	1.71 (1.67-1.75)	3.82 (3.68-3.96)	3.43 (3.36-3.50)	1.51 (1.46-1.56)
Austria	1.52 (1.45-1.59)	1.07 (0.97-1.16)	1.22 (1.18-1.26)	1.09 (0.99-1.18)
NL, 2	1.06 (1.02-1.11)	1.41 (1.26-1.55)	1.35 (1.21-1.48)	1.18 (1.11-1.25)
Greece	0.94 (0.88-1.00)	0.83 (0.78-0.87)	1.40 (1.33-1.47)	1.13 (1.08-1.18)
Germany	0.94 (0.88-1.00)	0.91 (0.79-1.03)	1.16 (1.05-1.27)	0.66 (0.58-0.73)
NL, 1	0.90 (0.84-0.97)	0.99 (0.82-1.17)	1.05 (0.82-1.27)	1.17 (1.07-1.26)
UK, 1	0.73 (0.68-0.77)	0.61 (0.45-0.76)	0.69 (0.63-0.76)	0.82 (0.74-0.89)
Spain	0.71 (0.67-0.75)	1.06 (1.01-1.10)	0.57 (0.52-0.62)	0.72 (0.66-0.79)
UK, 2	0.70 (0.67-0.73)	0.60 (0.55-0.65)	0.38 (0.36-0.41)	0.69 (0.62-0.75)
NL, 3	0.63 (0.56-0.71)	1.11 (0.87-1.35)	1.06 (0.73-1.39)	1.03 (0.82-1.23)
Latvia	0.54 (0.52-0.55)	0.42 (0.40-0.44)	0.35 (0.33-0.36)	0.40 (0.36-0.43)
Slovenia	0.50 (0.48-0.52)	0.48 (0.44-0.53)	0.59 (0.54-0.65)	0.96 (0.88-1.05)

Scale

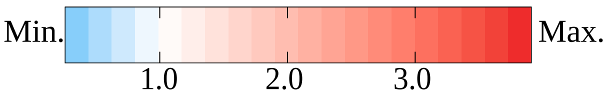

Supplement: S10 Text — (PDF) [file pmed.1003208.s013.pdf]
