# Supplementary material for: Variation in antibiotic prescription rates in febrile children presenting to emergency departments across Europe (MOFICHE): A multicentre observational study
Source: PLoS Med. 2020 Aug 19;17(8):e1003208. doi: 10.1371/journal.pmed.1003208 (PMC7444592; doi:10.1371/journal.pmed.1003208)

S3 Fig - Heat map of standardized broad-spectrum vs narrow-spectrum rates for viral, bacterial or unknown bacterial/viral infections (95% CI)

All adjusted for age, gender, season, comorbidity, fever duration, warning signs, CRP, focus of infection and previous antibiotic use.
*Also adjusted for triage urgency and focus of infection

EDs	**Viral*** n=1399	**Bacterial*** n=7365	**Unknown bacterial/viral** n=2306
UK, 3	1.54 (1.51-1.57)	1.86 (1.83-1.89)	1.64 (1.61-1.68)
Greece	1.15 (1.11-1.20)	1.08 (1.03-1.12)	0.86 (0.84-0.89)
NL, 2	1.07 (1.02-1.11)	1.10 (1.07-1.13)	1.09 (1.04-1.14)
NL, 1	0.97 (0.89-1.05)	1.04 (0.99-1.08)	1.13 (1.07-1.19)
Spain	0.96 (0.90-1.03)	0.98 (0.95-1.01)	0.94 (0.84-1.04)
UK, 1	0.96 (0.91-1.01)	0.81 (0.78-0.85)	0.56 (0.53-0.60)
NL, 3	0.95 (0.85-1.04)	0.94 (0.88-1.01)	0.84 (0.74-0.94)
Austria	0.94 (0.88-1.00)	1.21 (1.18-1.24)	1.05 (1.00-1.10)
Germany	0.93 (0.84-1.02)	0.95 (0.90-0.99)	0.98 (0.89-1.07)
UK, 2	0.92 (0.88-0.97)	0.77 (0.75-0.80)	0.44 (0.42-0.46)
Slovenia	0.76 (0.69-0.83)	0.69 (0.67-0.70)	0.93 (0.79-1.08)
Latvia	0.57 (0.48-0.67)	0.66 (0.65-0.67)	0.81 (0.67-0.94)

Scale

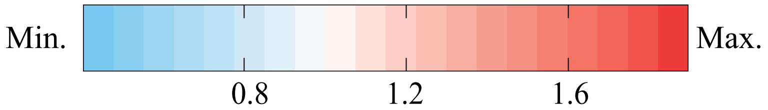

Supplement: S3 Fig — (PDF) [file pmed.1003208.s003.pdf]
