# Supplementary material for: Variation in antibiotic prescription rates in febrile children presenting to emergency departments across Europe (MOFICHE): A multicentre observational study
Source: PLoS Med. 2020 Aug 19;17(8):e1003208. doi: 10.1371/journal.pmed.1003208 (PMC7444592; doi:10.1371/journal.pmed.1003208)
Supplement: Attachment — Submitted filename: 20200228 ReplytoReviewers_final.docx [file pmed.1003208.s015.docx]

Manuscript reference number: PMEDICINE-D-19-04008

**Title: Variation in antibiotic prescription rates in febrile children presenting to Emergency Departments across Europe**

Dear Louise Gaynor-Brook,

Thank you for reviewing the paper entitled: “Variation in antibiotic prescription rates in febrile children presenting to Emergency”. We are very pleased to hear that our manuscript is considered for publication in PLOS Medicine. We have improved our manuscript according to the recommendations and taken this opportunity to clarify some issues raised by the editors and reviewers.

Foremost, the reviewers acknowledge the importance of the studied topic and our large detailed dataset including the classification of bacterial and viral disease in children with fever attending European EDs. In addition, they indicate the relevance of our findings of large variation of antibiotic prescription rates and broad-spectrum prescription rates in different EDs, and high variation of antibiotic prescription rates in viral infections and infections of unknown cause. We thank the editor and reviewers for thoroughly reviewing the manuscript and their comments to improve the paper. Additional analyses were performed as indicated by the reviewers. We feel that we were able to address the concerns and comments in the revised version and the manuscript has significantly improved; our response to the individual review items can be found below.

-----------------------------------------------------------

# Requests from the editors:

1) General comments: Please replace ‘gender’ with ‘sex’ throughout the manuscript .

Please position reference brackets before any punctuation (comma or full stop), separated by a space.

**Answer 1:** We have replaced gender with sex throughout the manuscript and adjusted the reference brackets.

**2)** Please revise your title according to PLOS Medicine's style. We suggest “Variation in antibiotic prescription rates in febrile children presenting to Emergency Departments across Europe (PERFORM): a multicentre observational study”

**Answer 2:** We thank the editors for this suggestion and adjusted the title accordingly.

**3)** Data Availability: PLOS Medicine requires that the de-identified data underlying the specific results in a published article be made available, without restrictions on access, in a public repository or as Supporting Information at the time of article publication, provided it is legal and ethical to do so. Please see the policy at <https://eur01.safelinks.protection.outlook.com/?url=http%3A%2F%2Fjournals.plos.org%2Fplosmedicine%2Fs%2Fdata-availability&amp;data=02%7C01%7Ch.a.moll%40erasmusmc.nl%7Cd3d3e012c87542422b9e08d79823ef48%7C526638ba6af34b0fa532a1a511f4ac80%7C0%7C0%7C637145152492405072&amp;sdata=9nDgUzRp4NURtFzu5%2BtW3Wwbkmd6flgyCjt267%2BK6nQ%3D&amp;reserved=0> and FAQs at

<https://eur01.safelinks.protection.outlook.com/?url=http%3A%2F%2Fjournals.plos.org%2Fplosmedicine%2Fs%2Fdata-availability%23loc-faqs-for-data-policy&amp;data=02%7C01%7Ch.a.moll%40erasmusmc.nl%7Cd3d3e012c87542422b9e08d79823ef48%7C526638ba6af34b0fa532a1a511f4ac80%7C0%7C0%7C637145152492405072&amp;sdata=7m6mGyVSkvVf41SMBVZl2A8ZGJgCrzqvoaQHwvXGAFk%3D&amp;reserved=0>

If the data are not freely available, please describe briefly the ethical, legal, or contractual restriction that prevents you from sharing it. Please also include an appropriate contact (web or email address) for inquiries (please note that this cannot be a study author).

**Answer 3:** A data set containing individual participant data will be made available in a public data repository containing a specific DOI. The data will be anonymized and will not contain any identifiable data. The data manager of the PERFORM consortium can be contacted for inquiries ([Tisham.de08@imperial.ac.uk](mailto:Tisham.de08@imperial.ac.uk)).

**4)** Abstract Background: Provide expand upon the context of why the study is important. The final sentence should clearly state the study question. Please define ‘PERFORM’

**Answer 4:** We agree with the editor that the importance of the study needs more clarification.

We have adjusted these sentences in the Abstract Background:

**“Background** Prescription rate of antibiotics is high for febrile children visiting the Emergency Department (ED), contributing to antimicrobial resistance*. Large studies at European EDs covering diversity in antibiotic and broad-spectrum prescriptions in all febrile children are lacking. A better understanding of variability in antibiotic prescriptions in EDs and its relation with viral or bacterial disease, is essential for the development and implementation of interventions to optimize antibiotic use.* As part of the PERFORM project *(Personalized Risk assessment in Febrile illness to Optimize Real-life Management across the European Union,* [*http://www.perform2020.org*](http://www.perform2020.org)*)*, the MOFICHE study (Management and Outcome of Fever in Children in Europe) aims to *investigate variation and appropriateness* of antibiotic prescription in febrile children visiting EDs in Europe. “

**5)** Abstract Methods and Findings:

Please provide more detail on the setting (e.g. which European countries, types of hospital) and brief demographic details on the study population (e.g. age, sex)

In the last sentence of the Abstract Methods and Findings section, please describe the main limitation(s) of the study's methodology.

**Answer 5:** We thank the editor for these comments and provided more details on the settings and demographic details of the study population in the abstract. In addition, we have adjusted the main limitations of the methodology of the study.

“Between January 2017-April 2018 data were prospectively collected on febrile children aged 0-18 years presenting to 12 EDs *in 8 European countries (Austria, Germany, Greece, Latvia, the Netherlands (n=3), Spain, Slovenia, United Kingdom (n=3)). These EDs were based in university (n=9) or large teaching hospitals (n=3).* Main outcomes *were proportion of* antibiotic prescription per *number of patients per ED, and among those with antibiotic prescription, the proportion of* broad-spectrum antibiotics.”

“In this analysis of 35,650 children *(median age 2.8 years, 55% male),* antibiotic prescription rates ranged between 22.4-41.6%, and among those broad-spectrum antibiotics rates ranged between 33.0-90.3%.”

*“A limitation of our study is that the included EDs are not representative for all febrile children attending the EDs in that country.”*

Please begin your Abstract Conclusions with "In this study, we observed ..." or similar.

Please interpret the study based on the results presented in the abstract, emphasizing what is new.

**Answer 6:** The Abstract Conclusions sections was adjusted following these useful recommendations.

**“Conclusions** In this study, we observed wide variation between European EDs of prescriptions of antibiotics and broad-spectrum antibiotics in febrile children. Although minor variation was observed in bacterial infection, great variation was present in viral infections and infections of unknown cause. Overall, one third of prescriptions were inappropriate or inconclusive with marked variation between EDs. In respiratory and urinary infections, guideline-concordance of prescriptions varied widely across EDs. Until better diagnostics are available to accurately differentiate between bacterial and viral aetiologies, implementation of antimicrobial stewardship guidelines is urgently needed across Europe.”

**7)** At this stage, we ask that you include a short, non-technical Author Summary of your research to make findings accessible to a wide audience that includes both scientists and non-scientists. The Author Summary should immediately follow the Abstract in your revised manuscript. This text is subject to editorial change and should be distinct from the scientific abstract. Please see our author guidelines for more information: <https://eur01.safelinks.protection.outlook.com/?url=https%3A%2F%2Fjournals.plos.org%2Fplosmedicine%2Fs%2Frevising-your-manuscript%23loc-author-summary&amp;data=02%7C01%7Ch.a.moll%40erasmusmc.nl%7Cd3d3e012c87542422b9e08d79823ef48%7C526638ba6af34b0fa532a1a511f4ac80%7C0%7C0%7C637145152492405072&amp;sdata=uT9hgl2ujaND1j%2FECB7TopLlHJJkjPmn%2BLr88BfFmb4%3D&amp;reserved=0>

**Answer 7:** We have added the Author Summary in the revised manuscript.

*“Why Was This Study Done?*

- *Respiratory infections, which are mainly caused by viruses, account for the majority of antibiotic use in children. In children with respiratory infections, antibiotic prescriptions varies across emergency departments (EDs) in Europe.*
- *Variation of antibiotic prescription and its relation with viral or bacterial disease is unknown for all children with fever at the ED.*
- *In order to optimize antibiotic prescriptions, it is important to better understand variability and appropriateness in antibiotic prescriptions.*

*What Did the Researchers Do and Find?*

- *In this prospective observational study, we included routine information of 35,650 children (median age 2.8 years) with fever attending 12 different EDs in Europe and calculated the proportion of antibiotic prescriptions and broad-spectrum antibiotic prescriptions. We adjusted for differences in population including age, comorbidity, disease severity, and focus and cause of infection.*
- *Across EDs, antibiotic prescription rates ranged between 22.4-41.6%, and of these prescriptions, broad-spectrum antibiotics rates ranged between 33.0-90.3%. Standardized antibiotic prescription rates ranged between 0.77 and 1.35 and standardized rates of broad-spectrum antibiotics ranged between 0.65-1.75.*
- *Standardized antibiotic rates varied in viral infections (0.1 to 3.3), infections of unknown cause (0.1 to 1.8). Reassuringly, in bacterial infections, antibiotic prescription was comparable between EDs (0.9 to 1.1).*
- *Prescription which were inappropriately indicated in ranged 0.6-29.3%), inconclusive prescriptions ranged 0.5 -61.7% and oral prescriptions of inappropriate duration ranged 4.4-59.0%). Prescriptions which were not concordant to the guideline ranged 11.8-47.3% in uncomplicated respiratory tract infections and ranged 11.1-100% in uncomplicated urinary tract infections.*

*What Do These Findings Mean?*

- *Prescription of antibiotics and broad-spectrum antibiotics varies between EDs in children with fever even corrected for age, comorbidity, disease severity, diagnostics, and focus and cause of infection.*
- *Variation was especially large in viral infections and infections of unknown cause.*
- *In all febrile children, one third of prescriptions were of inappropriate or inconclusive indication with variation between EDs. In addition, guideline-concordance for respiratory and urinary infections varied widely across EDs.*
- *Generalization of these results to all EDs in Europe, should be undertaken with caution. Implementation of guidelines is needed to reduce inappropriate prescription of antibiotics, whilst new biomarkers will further improve antibiotic prescription.*

**8)** Please expand upon your Introduction to address past research and explain the need for and potential importance of your study. Indicate whether your study is novel and how you determined that. If there has been a systematic review of the evidence related to your study (or you have conducted one), please refer to and reference that review and indicate whether it supports the need for your study.

Line 123 - please correct ‘ED’s’ to ‘EDs’

**Answer 8:** We have customized the introduction in order to support the importance of our study and the related evidence.

“*A literature review on antibiotic prescriptions and its determinants in febrile children in emergency care found large heterogeneity of studied populations which limited the ability to draw conclusions [1].* One recent European study, focusing solely on EDs, showed significant differences in antibiotic prescription rates in otherwise healthy children with respiratory tract infections [2]. Large studies at EDs across Europe *are lacking* that cover antibiotic and broad-spectrum prescriptions in all febrile children, *including patients with comorbidity and different diagnostic groups.* *Additionally, previous studies have addressed appropriate prescribing on diagnosis based on coded of the International Classification of Diseases [3-5]. This classification, however, may not accurately take into account bacterial or viral aetiology. Antibiotic prescription rates for viral and bacterial disease using a structured classification have not yet been investigated at EDs.*

A better understanding of variability in antibiotic prescriptions in EDs *and its relation with bacterial or viral disease,* taking into account differences in case-mix, is essential for the development and implementation of interventions to optimize antibiotic use.”

We have corrected ED’s to EDs.

**9)** Methods

Thank you providing a STROBE checklist. Please add the following statement, or similar, to the Methods: "This study is reported as per the Strengthening the Reporting of Observational Studies in Epidemiology (STROBE) guideline (S1 Checklist)." When completing the checklist, please use section and paragraph numbers, rather than page numbers.

**Answer 9:** We have added this statement at the end of the Methods section. In addition, we have used sections and paragraph numbers instead of page numbers.

*“This study is reported as per the Strengthening the Reporting of Observational Studies in Epidemiology (STROBE) guideline (S1 Checklist)."*

**10)** Did your study have a prospective protocol or analysis plan? Please state this (either way) early in the Methods section. If a prospective analysis plan was used in designing the study, please include the relevant prospectively written document with your revised manuscript as a Supporting Information file to be published alongside your study, and cite it in the Methods section. A legend for this file should be included at the end of your manuscript. If no such document exists, please make sure that the Methods section transparently describes when analyses were planned, and when/why any data-driven changes to analyses took place.

**Answer 10:** This study was a prospectively planned analysis of the MOFICHE database and a prospective analysis plan is available. First, pilot analyses were performed halfway of data collection (November 2017) and final analyses upon the complete data set were performed in December/January 2018. The analysis on antimicrobial resistance and relation with broad-spectrum rates were not pre-planned and were added in April 2019 after the first data analysis. We have clarified the timing of the analyses in the Statistical Analysis Plan. The prospective analysis plan is added as a Supporting information file and is cited in the Methods section in the Data analysis section.

*“This is a main analysis of the MOFICHE study (Management and Outcome of Febrile children), which is embedded in the PERFORM project (Personalized Risk assessment in Febrile illness to Optimize Real-life Management across the European Union) [6].”*

*“The data were analysed using an a priori statistical analysis plan (S3 appendix).”*

**11)** Results

Throughout your results, please indicate what the results in brackets represent (mean? and range)

Line 326-328: Please provide 95% CIs and p values

Lines 337 & 443 - Please indicate which factors are adjusted for

Line 356 - please clarify what is meant by ‘prescription rates remained’

Line 359 - please correct to ‘upper respiratory tract infection’

**Answer 11:** We have clarified the results sections according to his comments.

Line 325-328: “Higher standardized rates for broad-spectrum antibiotics were not related to higher antimicrobial resistance percentages on a national level (resistance S. aureus ((ρ -0.07 95% CI (-0.62 to 0.53), p=0.83), S. pneumoniae (ρ -0.31 95% CI (-0.77 to 0.35), p=0.35) and E. coli (ρ 0.07 95%CI (-0.52 to 0.62), p=0.82) or on a hospital level (methicillin resistance rates for S. aureus ρ 0.12 (95% CI (-0.48 to 0.65), p=0.70) (S5 appendix).”

Line 337: “*Adjusted for general characteristics, disease severity, diagnostics and focus of infection,* we observed large…”

Line 443: “*After adjustment of general characteristics, disease severity, diagnostics and focus and cause of infection,* we observed variability of both antibiotic and broad-spectrum prescriptions in respiratory tract infections, variability in antibiotic prescriptions in viral and unknown infections, and variability of broad-spectrum use in all diagnostic groups.”

Line 356: “*After adjustment of general characteristics, disease severity, CRP and cause of infection, variability remained in antibiotic prescription rates* for patients with lower respiratory tract infection”

Line 359:“..*and other upper respiratory tract infections*”

**12)** At line 374, you describe the study as a "large prospective multicentre study". It may be that the study reports a retrospective analysis of prospectively collected data, and we ask you to adapt the language as appropriate.

**Answer 12:** This study is a prospectively planned analysis of prospectively collected data. This study is a main part of the PERFORM project (<https://www.perform2020.org>, <https://clinicaltrials.gov/ct2/show/NCT03502993>). In the preparation phase of the study, we decided on the main outcomes of the study: antibiotic prescription rate and broad-spectrum antibiotic prescription rate. After this preparation phase, we started data collection. In our opinion, the term large prospective multicentre study is appropriate.

**13)** Discussion

Please remove subheadings within the Discussion section

Please present and organize the Discussion as follows: a short, clear summary of the article's findings; what the study adds to existing research and where and why the results may differ from previous research; strengths and limitations of the study; implications and next steps for research, clinical practice, and/or public policy; one-paragraph conclusion.

**Answer 13:** We have removed the subheadings in the Discussion section and organized the discussion following these recommendations.

**14)** References

Please ensure that journal titles are appropriately formatted and capitalised e.g. ref 7 BMJ; ref 22 JAMA; ref 43 formatting issues

Please add additional access details to reference 16.

Can the title of reference 43 be translated?

**Answer 14:** We have changed the references following these guidelines.

Journal titles are formatted and capitalised, access details are added to reference 16 and we translated the tile of reference 43.

**15)** Supplementary Files

S2 Appendix - ‘investigation’ is misspelt in the figure

S3 Appendix - please correct ref 5

**Answer 15:** Investigation is corrected in the figure (Fig 1) and in ref 5 bmj is capitalised to BMJ.

# Comments from the reviewers:

## Reviewer #1:

**1.1)** This descriptive, cross-sectional study documents widespread variation in antibiotic prescribing among 12 European emergency departments to children who either present with fever or had a recent history of fever. The authors find widespread variation in prescribing particularly for viral conditions or conditions in which the etiology is either viral of bacterial.

The authors have collected a large, rich, and impressive set of clinical data - by far the greatest strength of this paper. Moreover, there is no question that antibiotic stewardship in the ED for children is of utmost importance. The manuscript is generally easy to follow and the figures are helpful, particularly the Appendix figure with a flow chart with how the various categories like "presumed bacterial" were defined.

Despite these strengths, my major concern is that the research question the authors have asked - i.e., what is the degree of variation in antibiotic prescribing among these 12 European EDs - seems like the least interesting question they could have addressed using their impressive data. Based on the way it is framed, this study runs the risk of being seen as another paper documenting variation in care among some defined group of units (in this case, EDs). The weaknesses of such papers are 1) So many similar papers have been published that the only truly shocking finding would be the LACK of variation in care; and 2) It is difficult to know exactly to know what to do when variation is demonstrated, as the "appropriate" amount of variation is unknown even when you can adjust for the rich set of clinical variables that the authors have access to. This is the Achilles heel of variations-based analyses - at best, they can hint at potential overuse of care (in this case,

antibiotics).

**Answer 1.1:** We thank the reviewer for his thorough review and important and useful suggestions to improve the paper. We agree there are multiple papers addressing variation of antibiotic prescribing. Although these variations-based analysis can only hint at potential overuse of antibiotics, we do think our study is an important addition to the current literature. First, unlike other studies, we have adjusted for important prospectively collected confounders including hospital-level characteristics and patient-level characteristics including alarming signs, disease severity, working diagnosis and aetiology of infection. Second, our structured classification of bacterial and viral disease enabled us to perform more detailed analysis in groups where antibiotics use is likely appropriate or likely inappropriate as suggested. Other studies that address appropriate and inappropriate prescribing of antibiotics focus on diagnoses based on ICD codes, which lack important details on aetiology of infections and adjustment for confounders. Third, most existing studies either cover primary care or hospital settings, and do not address antibiotic prescriptions in emergency care. We agree with the reviewer this needs more clarification in the paper and therefore we have adjusted the Introduction, added extra analyses to categorize appropriate and inappropriate use of antibiotics in the different EDs and the discussion of the paper.

Some of the more important questions that could have been asked with these rich data include:

**1.2)** Why is there so much apparent over-testing? 45% of the children received a CRP - perhaps this is just a trans-Atlantic difference, but CRP is not routinely obtained for febrile children in the ED in the U.S., nor is it viewed as a particularly useful discriminator between viral and bacterial infections. Similarly, 25% of children received a urinalysis, but the focus of infection was the urinary tract for just 3.5% of children. These numbers boggle my mind.

**Answer 1.2:** We think the use of CRP in Europe might indeed be different compared to North America. In Europe, CRP measurement is commonly used at the ED in children. Based on a systematic review [7] CRP out performs white blood cell count or neutrophil count. In several multicentre studies CRP showed discriminative value for viral and bacterial infection [8-10].

In children, urinary tract infections can present as fever without focus or with aspecific symptoms.

The number of tests of urinalysis are higher in order to detect one child with a urinary tract infection. Indeed, 25% of children received a urinalysis, and 3.5% of children were classified as urinary tract infection.

**1.3)** What is the estimated rate of antibiotic overuse? The authors could devise a system, for example, in which they consider antibiotics to be likely inappropriate if the patient had a confirmed or probable viral infection; likely appropriate if there was a confirmed or probable bacterial infection; and possibly appropriate for everything else. They could use their rich data on lab results, CXR results, and diagnoses to further their classification scheme. They could then perhaps utilize the variation between EDs to establish achievable benchmarks of prescribing for the "possibly appropriate" category (e.g., the 20th percentile of prescribing).

**1.4)** Given that the authors presumably have data on prescription duration and whether the agent was broad spectrum vs not, they could go even further and classify not just whether the indication was antibiotic-appropriate or not, but also whether the duration and choice of antibiotic were appropriate. They could calculate the proportion of antibiotics prescribed that had any deficit (inappropriate indication, inappropriate duration, inappropriate agent). These are questions that are really difficult to answer using the administrative datasets that most studies of antibiotic prescribing rely on.

My point is simply that demonstrating variation alone does not move the needle very much, because there will always be variation. It is simply a shame in my mind to use the amazing data the authors have collected simply to document the existence of variation.

**Answer 1.3 and 1.4:** We thank the reviewer for this important comment and the excellent suggestion for classifying antibiotic prescription in likely appropriate/likely inappropriate. We have added extra analyses to the paper classifying antibiotic prescriptions as ‘likely appropriate’ (definite bacterial, probable bacterial, bacterial syndrome), ‘likely inappropriate’ (definite viral, probable viral, viral syndrome) and inconclusive (unknown bacterial/viral) according to the flowchart of the aetiology of infection. We have provided results for proportions of overall appropriateness of prescriptions and specified this for each ED separately. In addition, we added the proportion of inappropriate duration. Furthermore, we added the proportion of guideline concordant prescribing (appropriate agent) for upper and lower respiratory tract infections and urinary tract infections. The proportion of antibiotic that had any deficit (inappropriate indication, inappropriate duration) were assessed in all oral prescriptions. In respiratory tract infections and urinary tract infections we assessed appropriateness at three measures: appropriateness of indication, appropriate duration and guideline-concordance. We have changed the Abstract, Methods, Results and Discussion section accordingly:

Abstract:

Methods and Findings:

*“In all febrile children, antibiotic prescriptions were appropriate in 65.0%, inappropriate in 12.5% (range EDs: 0.6-29.3%) and inconclusive in 22.5% (range EDs: 0.4-60.8%). Prescriptions which were not concordant to the guideline ranged 11.8-47.3% in uncomplicated RTI and ranged 11.1-100% in uncomplicated urinary tract infections.”*

Main text:

Methods:

*“We estimated appropriateness of antibiotic prescriptions using three estimates: appropriate indication, appropriate duration and, for respiratory and urinary tract infections, guideline-concordance. For appropriate indication, we calculated the proportion of antibiotic prescriptions that were ‘likely appropriate’, ‘likely inappropriate’ and ‘inconclusive’ of all antibiotic prescriptions. Antibiotic prescriptions were classified as ‘likely appropriate’ in presumed bacterial infections (definite bacterial, probable bacterial, bacterial syndrome), ‘likely inappropriate’ in prescriptions in presumed viral infections (definite viral, probable viral, viral syndrome), and ‘inconclusive’ in prescriptions in unknown bacterial/viral infections or others. Second, we estimated the proportion of oral prescriptions that were of inappropriate duration. Inappropriate duration was defined as >10 days for treatment of tonsillitis with beta-lactamase sensitive penicillins (J01CE) and >7 days for all other prescriptions according to recommendations by international guidelines [11-13]. For all oral prescriptions, we calculated the number of prescriptions that were both inappropriate in indication (‘likely inappropriate’) and in duration, and the number of prescriptions that were either inappropriate in indication or inappropriate in duration.*

*In addition, for oral prescription in patients with uncomplicated respiratory tract infections and uncomplicated urinary tract infections, we calculated the proportion of prescribed antibiotic types that matched the antibiotic type advised in the local guideline (‘guideline-concordant prescription’) (S5 appendix). Uncomplicated infections were defined as infections in previously healthy children who did not receive therapeutic antibiotic treatment before ED visit. For all oral prescriptions in uncomplicated respiratory tract and urinary tract infections, we calculated the number of prescriptions that were inappropriate for all the three estimates (indication, duration, guideline-concordance), and the number of prescriptions that were inappropriate in any of the three groups.”*

Results:

*“In all antibiotic prescriptions, 65.0% (7386/11371) were estimated as likely appropriate, 12.5% (1418/11371) were likely inappropriate and 22.5% (2567/11371) were inconclusive (Fig. 8). Oral antibiotic prescriptions with inappropriate duration were found in 19.9% (1525/7636). Of all oral antibiotic prescriptions, 2.1% (134/7636) were both of inappropriate indication and inappropriate duration, whereas 30.0% (2294/7636) was either of inappropriate indication or of inappropriate*

*Across EDs, likely appropriate prescriptions ranged 23.7 to 98.9%, likely inappropriate prescriptions ranged 0.6 to 29.3%, inconclusive prescriptions ranged 0.5 to 61.7%, and oral prescriptions with inappropriate duration ranged 4.4 to 59.0% (Fig. 8). Oral prescriptions with both inappropriate indication and duration ranged 0.0 to 8.4% and prescriptions with either inappropriate indication or duration ranged 11.3 to 69.9%.*

*In uncomplicated respiratory tract infections, oral prescriptions were not guideline-concordant in 22.3% (973/4373). In this group, proportion of prescriptions that were inappropriate in all three estimates (indication, duration, guideline-concordance)* was 0.7% (31/4373), whilst 48.8% (2136/4373) were inappropriate in any of the three estimates. In uncomplicated urinary tract infections, oral prescriptions were not concordant to the guideline in 45.1% (152/337) and 65.9% (222/337) were inappropriate in any of the three estimates. Across EDs, inappropriateness in any of the three estimates ranged 15.7-80.9% for respiratory tract infections and 11.1-100% for urinary tract infections.”

Discussion:

*“Moreover, one third of all antibiotic prescriptions were of inappropriate or inconclusive indication, and 20% of oral prescriptions were of inappropriate duration with large variation across EDs. Between EDs, oral prescriptions that were not concordant to the guideline varied from 12-47% in respiratory tract infections and varied from 11-100% in urinary tract infections.”*

*“Previous studies in the United States have evaluated appropriateness of antibiotic prescribing in children defined by ICD codes. Poole et al. found that prescriptions were in general not indicated in 32% of emergency care visits in children. Additionally, overall prescription of first-line antibiotics (amoxicillin, amoxicillin-clavulanate) ranged 50-78% for respiratory tract infections in children [14-16]. We found a similar rate of guideline concordant prescriptions for respiratory tract infections (78%), whilst guideline concordance was defined differently for most EDs: amoxicillin and small-spectrum penicillins according to the local guideline. One ED (UK, 3) used amoxicillin-clavulanate as first-choice for respiratory tract infections. Our study is the first to evaluate appropriateness of antibiotic prescribing in febrile children visiting different EDs in Europe, using a structured flowchart categorizing viral, bacterial and unclassified infections, and taking local guidelines into account.”*

*“Appropriate antibiotic prescribing exists of different features such as indication, route of administration, duration and guideline-concordance. In our study, we did not observe a clear association between inappropriate indicated prescriptions and prescriptions of inappropriate duration. This indicates that guideline implementations should focus on these different aspects of appropriate antibiotic prescribing.”*

Conclusion:

*“Furthermore, indications of prescriptions were inappropriate or inconclusive in one third of prescriptions and varied between EDs. In respiratory and urinary infections, guideline-concordance of prescriptions varied widely across EDs.”*

Other issues

**1.5)**	Why focus only on children with fever or a history of fever? Many antibiotics are inappropriately written to children to afebrile children with, say, a 10-day course of non-improving cough.

**Answer 1.5:** We agree antibiotics should be prescribed cautiously in afebrile children. However, in this study, we have focused on children with fever. We also included children with a history of fever as antipyretics is commonly used by parents and therefor these children might present without high temperature due to antipyretics.

**1.6)**	The authors say that they ran a multilevel logistic regression model with clustering on the hospitals. Those two terms don't go together - one typically can either regarding clustering as a nuisance and adjust for it (e.g., generalized estimating equations) vs view the variation between clusters as being of primary interest (multilevel model). I'm pretty sure what the authors did is run a multilevel logistic model with a random hospital intercept (different from clustering on hospital)", but this should be clarified.

**Answer 1.6:** We apologize for not being clear on the model. We used a multilevel model with a random intercept for each ED. We have clarified this in the data analysis section.

Methods Data Analysis:

*“Second, we used multilevel logistic regression with a random intercept for each ED to study variation of antibiotic use between hospitals.”*

**1.7)**	I can't tell if this is in the model or not, but one of the patient-level predictors should be current fever in the ED vs no fever but positive history - clinicians are definitely more likely to prescribe antibiotics if the child has a fever in the ED. On that note, please write out the regression model - it is not clear from the text what variables are included or not.

**Answer 1.7:** We thank the reviewer for this excellent suggestion and agree this is an important predictor to add to the model. We have added this in the model and adjusted the methods and results accordingly. Furthermore, we specified the regression model in more detail in a table and in a supplemental file S4 appendix.

Methods:

“Patient-level factors were selected a-priori and included general characteristics (age, sex, season, comorbidity, referral (referred vs self-referred), markers for disease severity as triage urgency (high urgency (immediate, very urgent, intermediate) versus low urgency (standard, non-urgent)), fever duration in days, *fever measured at ED visit (>38 C)* and presence of NICE guideline red warning signs (0, 1, ≥2).”

*“Specifications of the adjusted model are presented in table 1 and in S4 appendix.”*

Results:

“After correction for….., variability for antibiotic prescriptions remained between EDs in the adjusted model (range standardized prescriptions rates: *0.77-1.35*, MOR 2.41). Variation was also observed for intravenous vs oral administration (range standardized rates 0.29-1.31, *MOR 2.60*) …”

**1.8)**	I think it would be more sensible to restrict the sample only to treat-and-release ED patients - examining antibiotics started in the ED for hospitalized patients seems a bit odd.

**Answer 1.8:** To enable to provide an unbiased proportion of antibiotic prescription, we have focused on including patients based on presenting symptoms and not based on the decision to admit. Furthermore, EDs have different criteria to admit patients and variation exists in hospital admission across EDs [17] and selection on admittance might bias the results. Patients can also be admitted for oxygen therapy or monitoring, and not necessary for antibiotic treatment.

## Reviewer #2:

Thank you for the opportunity to read and review with interest the results of the PERFORM study, a multi-centre observational study describing antibiotic prescribing rates for children < 18 years across several large teaching hospitals in Europe. The study is largely descriptive in nature, quantifying variation in prescribing across hospital centres, and describing the nature of the prescribing behaviour (by type) and consideration of patient and hospital factors which be associated with prescribing rates. The results are topical as growing antibiotic resistance is a continuing problem in many health care systems and reports such as these are very helpful is quantifying the scale of the issue. I have a few comments in regards to mostly the methodology and some suggestions which the authors may want to consider:

To help improve clarity in abstract reporting:

**2.1)**	Methods - when describing primary outcomes, it is unclear what the denominator is in the rates and what type of unit of measurement this is: i.e. general prevalence as a %, per specific number. Please include how prescription rate is defined in the abstract.

**Answer 2.1:**

We thank the reviewer for reviewing our paper and the suggestions to improve the manuscript.

We realise that we are not clear on the outcome measures. We calculated the proportion of antibiotic prescriptions and among those with antibiotic prescription, proportion of broad-spectrum prescriptions per number of patients per ED.

We have adjusted the Abstract Methods accordingly:

*“Main outcomes were proportion of antibiotic prescription per number of patients per ED, and among those with antibiotic prescription, the proportion of broad-spectrum antibiotics.*

**2.2)**	Findings - "after standardisation" - It would be helpful to describe how rates were standardisation as there are several different methods of standardisation rates (i.e. direct/indirect)

**Answer 2.2:** We used indirect standardization, where the expected number of antibiotic prescriptions was standardized to the average ED.

We have added the following paragraph to the methods section to further explain how the standardized prescription rates were calculated:

*“We calculated standardized antibiotic prescription ratios using indirect standardization, where the expected number of antibiotic prescription was standardized to the average ED. Standardized antibiotic prescription ratios are the ratio between observed antibiotic prescriptions in an ED and the expected antibiotic prescriptions in an ED. The expected number of antibiotic prescriptions was estimated through the adjusted model, by summing the predicted probabilities from the adjusted model of antibiotic prescription for each of the patients. Standardized prescription rates >1 demonstrate higher prescriptions rates than expected and rates <1 indicate lower prescriptions rates than expected.”*

**2.3)**	Findings Line 92 - The abstracts reads as if broad and narrow spectrum antibiotics were supposed to be compared but the authors seem to report range combined. Please clarify

**Answer 2.3:** We studied two outcomes, namely antibiotic prescription rates (yes/no), and among those with antibiotic prescriptions we studies broad-spectrum prescriptions vs narrow-spectrum prescriptions. We have clarified this in the Abstract.

*“In this analysis of 35,650 children (median age 2.8 years, 55% male), antibiotic prescription rates ranged between 22.4-41.6%, and among those broad-spectrum antibiotics rates ranged between 33.0-90.3%. After standardization, differences in antibiotic prescriptions ranged from 0.8 to 1.4 and ratio between broad-spectrum and narrow-spectrum prescriptions ranged from 0.7 to 1.8 per ED.”*

**2.4)**	General comment on abstract findings reporting - Could the authors comment what constitutes descriptors such as "considerable variation", "varied substantially", and "little variation" as there is no a priori definition of this or explanation. I would generally favour refraining from using these adjectives without defining the range differences. For instance, in the results sections, the focus should be on just simply presenting the results (i.e "the range was: "). Instead, the qualitative interpretation of what constitutes "considerable", "little", variation should be provided in the context of the discussion. Alternatively, the authors wish to do this, they should define range definitions in the methods section.

**Answer 2.4:** We agree with the reviewer that these adjectives are not suitable for reporting findings. We have changed the Abstract and Results following these recommendations.

Abstract

*“Standardized antibiotic rates varied for viral infections (0.1 to 3.3), infections of unknown cause (0.1 to 1.8) and bacterial infections (0.9 to 1.1). Variation also exist for standardized antibiotic rates and broad-spectrum rates for lower and upper respiratory tract infections. ”*

Main text

Results:

*“Adjusted for general characteristics, disease severity, diagnostics and focus of infection, we observed variation for antibiotic prescriptions in viral infections (range standardized rate: 0.05-3.29, MOR 4.91)..”*

*“The proportion of broad-spectrum prescriptions varied for viral 38.9-91.4%, definite bacterial 39.2-96.0%, probable bacterial 28.5-86.3% and unknown bacterial/viral 20.0-95.7% (S9 appendix).”*

*“In particular, antibiotic prescriptions varied for otitis media (80.7%, range 39.8-92.5%) and tonsillitis/pharyngitis (51.3%, range 14.8-82.3%).”*

**In the main text:**

**2.5)**	Lines 151-152: This is quite important process of the study design - that was active data collection during the study period with training of research staff on data collection and clinical assessment. Hence, you could even argue there is an interventional component to this analyses and not purely an "observational" study. Whilst I recognise the rationale for this is to improve data capture and quality - the authors primary limitation is further amplified - that these hospitals may not necessary generalise because of this reason. What would have enhanced this would have been to have a pure retrospective one-year look-back period prior to the study active data collection taken place. Whilst it may not possible to do this, it is worth mentioning commenting in the discussion.

**Answer 2.5:** We agree with the reviewer there could have been a small interventional component in training the clinical and research staff. Although the suggestion of having a retrospective one year look-back could give more insight, unfortunately, this is not feasible. We trained clinical and research staff to improve data collection by creating more awareness of alarming signs and measurement of vital signs. Indeed, there is a balance between obtaining high quality data, optimizing data collection with low number of missing values versus influencing common practice in the data collection for an observational study. Although this could have slightly influenced compliance to reporting to guidelines, more awareness of alarming signs is not expected to influence AB prescription. We agree with the reviewer it is important to add this in the discussion section.

*“Our efforts to improve data quality by training clinical and research staff, might have influenced common clinical practice. Since this training focused on awareness of alarming signs in the clinical assessment of the febrile child, it is unlikely that it influenced antibiotic prescription.”*

**2.6)**	Lines 154: I'm am quite puzzled by the sample size justification. The rule-of-thumb of 10 events per variable sample size calculation generally is used as a justification for developing prognostic models. Besides the 10 EPV rule-of-thumb being outdated for prognostic models (see Riley et al. - <https://eur01.safelinks.protection.outlook.com/?url=https%3A%2F%2Fonlinelibrary.wiley.com%2Fdoi%2Ffull%2F10.1002%2Fsim.7992&amp;data=02%7C01%7Ch.a.moll%40erasmusmc.nl%7Cd3d3e012c87542422b9e08d79823ef48%7C526638ba6af34b0fa532a1a511f4ac80%7C0%7C0%7C637145152492405072&amp;sdata=TfZbPupuZeavQRvAQu3G6qBuadhurx4iOkuJlwSA6FY%3D&amp;reserved=0>), the author applied a multi-level model which means a design effect needs to accounted for (i.e. ICC between hospitals). Further, this study is also not testing a particular hypothesis as purely observational (primary outcome is simply a prescription rate) - so why not just determine the sample size based on the expected/desired precision on the prescribing rates?

**Answer 2.6**: We agree with the reviewer that the current sample size considerations are confusing. The sample size was estimated to be sufficient for the other aims of MOFICHE including other outcomes as hospitalization and blood culture rate.

In the study protocol the sample size estimation was defined as follows:

‘We expect to include nearly 40,000 children with at most 5% of missing data. Pilot data indicate on overall antibiotic prescription rate of 30%, hospitalization rate of 10%, a CRP rate of 15% and a blood culture rate of 5%. Applying a commonly used rule of thumb of 10 events per variable, we expect that the study sample is large enough to analyse at least 120 determinants for the most prevalent outcome of antibiotic prescription and 25 determinants for the least prevalent outcome of blood culture with sufficient accuracy.’

We agree with the reviewer that the current sample size considerations is confusing. We have therefore adjusted the sample size considerations to estimate a desired with of a 95% confidence interval of the estimated standardized prescription rates. The number of ED visits varied between 400-7000 with an event rate of approximately 30%. These numbers would allow the expected width of the confidence interval of the standardized prescription rates to be below 0.5 for the smallest ED. We have clarified this in the Methods section:

Methods:

*“We expected to include 40,000 children with at most 5% of missing data. Pilot data showed an overall antibiotic prescription rate of 30%. Applying 10 events per variable, this study is large enough to analyse over 1000 determinants for the outcome antibiotic prescription. We performed a post-hoc sample size estimation for a desired width of the 95% confidence interval (CI) of standardized prescription rates. The expected width of the CI of the standardized prescription rates was below 0.5 for the smallest ED.”*

**2.7)**	Lines 224: How many children were excluded with missing data on antibiotic use, presumed cause of infection, and focus of infection and did their characteristics differ from those who were included. It would be good to show if these children were similar or different to the analysis cohort.

**Answer 2.7:** We compared characteristics of children with missing data on antibiotic use, presumed cause of infection and focus infection, with patients with completed outcomes. We have added this analysis in a supplementary file and added it in the Results section.

“Of the total population of 38,480 patients, we excluded 738 *patients based on missing data of antibiotics or diagnosis,* and the repeated visit of 2,092 patients to the same ED. *Compared to patients with complete outcome data, patients with missing data were similar in age, sex, comorbidity and admission rates (S6 appendix).* “

**2.8)**	Lines 225-226: What was the rationale for including only the first visit for patients within the first five days? Is there any risk of under-ascertainment of prescribing rates if the initial visit did not include any prescribing but it subsequently occurred at subsequent visits?

**Answer 2.8:** We thank the reviewer for this question. The data collection did not include second visits for all patients as the sample period was one week period per month in some EDs. Inclusion of subsequent visits might bias our results due to missing follow-up data in some EDs. We added this in the Discussion.

*“Furthermore, we only included the 1^st^ visit of patients repeatedly visiting the ED since data collection did not include secondary visits in all EDs.*

**2.9)**	Lines 229: Use of multi-level logistic regression model needs some justification. For prescribing rates, you could also argue that a Poisson regression model and then incorporate patient level covariates in the model. This would essentially derive your rate over the period of time of data collection as well.

**Answer 2.9**: Since we collected pseudo-anonymous data, we were not able to match ED visits to individual patients. Furthermore, data collection did not include second visits for all patients as the sample period was one week period per month in some EDs. In this data, calculating the number of antibiotic prescriptions for individual patients over time is therefore not possible. Since logistic regression has less computational challenges than Poisson regression, we have found logistic regression to be more suitable.

**2.10)**	Lines 230-231: How were patient level factors selected for inclusion in the model. By a priori specification or was there any covariate testing for interaction and confounding. Please describe.

**Answer 2.10:** Patient level factors were chosen a priori. We included patient level factors that are known to be related with case-mix, disease severity and antibiotic prescription. We have clarified this in the Methods section.

*“*Patient-level factors *were selected a-priori according to literature [9, 18-21]…”*

**2.11)**	Lines 250-251: The standardisation process, what was the reference population used to obtain the expected antibiotic prescribing and where was this data obtained from?

**Answer 2.11:** We refer to comment 2.2 of reviewer 2 and have changed the methods section to more clearly describe the standardization procedure:

*“Standardized antibiotic prescription ratios are the ratio between observed antibiotic prescriptions in an ED and the expected antibiotic prescriptions in an ED. The expected number of antibiotic prescriptions was estimated through the adjusted model, by summing the predicted probabilities from the adjusted model of antibiotic prescription for each of the patients. Standardized prescription rates >1 demonstrate higher prescriptions rates than expected and rates <1 indicate lower prescriptions rates than expected.”*

**2.12)**	Lines 260-265: How did the authors define categories of "high" and "low" for their heat maps. What this based on evidence or consensus (i.e. planned analysis, post-hoc consensus). If would be useful to see the pre-specified analytical plan (if there was any) and if there wasn't, this also needs to be mentioned and commented in the discussion as a potential limitation.

**Answer 2.12:** The adjusted rates are calculated by the ratio of antibiotic prescription rates for the adjusted model with antibiotic prescription rates based on the model with only the intercept. In the heat maps ‘high’ categories are standardized rates >1 and ‘low’ categories are standardized rates <1. We have clarified this in the legends of the heat maps and have added the pre-specified statistical analysis plan as a supplemental file.

Figure legends:

*“EDs were ordered according to standardized antibiotic prescribing rates, from low to high on the left vertical axis. The coloured box represents rank of standardized rates for each ED; red colours indicate rates >1, blue colours indicate rates <1 and rates equal to 1 are white.”*

**2.13)**	Table 1, Final diagnosis is interesting as most look majority are labelled probable viral (57.2%), where definite bacterial only (4.1%) and probable bacterial is 18.1%. It would be useful if the authors could provide a metric of inappropriate or appropriate prescribing. This would require essentially mapping the prescription to the final diagnoses. This analysis stratified by hospital would be useful as Figure 3 shows the three UK hospital having the hospitals having the high rates of prescribing, but at the moment it's not conclusive whether high prescribing was due to having more patients with particular types of diagnoses.

**Answer 2.13:** We agree that final diagnosis is an important predictors for antibiotic prescription. Therefore, final diagnosis was already included as covariate in the adjusted analysis. We agree that more insight in final diagnosis and inappropriate and appropriate prescribing is important. As also suggested by reviewer #1, we have added analyses to estimate appropriate prescribing. We classified antibiotic prescriptions as “likely appropriate” (definite bacterial, probable bacterial, bacterial syndrome), “likely inappropriate” (definite viral, probable viral, viral syndrome) or “inconclusive” (unknown bacterial/viral, other) according to the final diagnosis. We have adjusted the methods and results sections accordingly: See also answer 1.3 and 1.4 to reviewer 1.

**2.14)**	Lines 325: Could this be due to the appropriateness of the prescribing. Can the authors comment on this?

**Answer 2.14:** The ED with highest standardized broad-spectrum rates (UK, 3) also had high proportion of inappropriate prescriptions and prescriptions that were disconcordant to the guideline for urinary tract infections. For respiratory tract infections, 19% of oral prescriptions was disconcordant to the guideline in UK, 3. UK, 3 was the only ED to have amoxicillin-clavulanate as first-choice agent for respiratory tract infections. We have added a heat map that showed proportion of prescriptions of inappropriate indication, inappropriate duration and disconcordance to the guideline per ED to provide more insight into the differences (figure 8). We agree this needs more clarification and therefore we have commented on this in the Discussion section. We also refer to answer 1.3 and 1.4 from reviewer 1 for other specific changes in Methods, Results and Discussion.

Discussion:

*“The ED with highest standardized broad-spectrum rate (UK, 3), did not have high inappropriate prescriptions for respiratory tract infections. In only this ED, amoxicillin-clavulanate (broad-spectrum) was first-choice agent for uncomplicated respiratory tract infections which could explain higher broad-spectrum rates in this ED.”*

## Reviewer #3:

This is a large prospective, multicenter observational study that assesses antibiotic prescription in children in 12 European EDs. Data collection was mostly based on routine clinical data, which makes this study particularly interesting. Since adequate disease classification for pediatric infectious diseases is not available for pediatric patients, the authors undertook the effort of classifying the likely etiology of infection. This study in an important first step towards more systematic data collection on antibiotic prescription in European ambulatory settings and provides important data on variability of antibiotic prescription practices. The authors should be congratulated on this important effort. The present manuscript focuses on patient-related factors to describe variations in the prescription rates observed. The manuscript would be greatly strengthened by taking more provider, health systems and data quality issues into account (or to at least give

the reader a sense to which extent these were taken into consideration). Specifically, the following points should be addressed further:

**3.1)**	This study relied on routine data collection. EDs are busy places with high staff turnover. I imagine that the EMR system varies a lot from hospital to hospital, as does the type and quality of routine medical documentation. One would like a better sense on how data was extracted from routine data sources and how the quality of this data was assessed? What are the influencing factors for data quality and completeness? The authors also mention that enumerators were trained in a CRF. I assume that not all routine providers were trained in data collection procedures for this study? How was the data extracted from the routine medical record system into the eCRF? How are antibiotic prescriptions recorded at the different sites? Are there differences in the completeness/ type of documentation between ambulatory and admitted patients? In some countries, for example, DRGs are implemented for admitted patients resulting in differential documentation for hospitalized patients due to billing issues.

**Answer 3.1:** We thank the reviewer for the thorough review and important suggestions to improve the paper. We agree that more explanation of the data collection should be provided.

- Before the start of data collection, we have inventoried local factors that influenced medical care at the ED including type of ED (mixed adults and paediatric, paediatric only), triage system (Manchester triage system, local system), supervision (direct supervision, indirect supervision, oversight), availability of general practitioner service, availability of general practitioner service during out-of-office hours and experience and background of physician in charge. Furthermore, we have inventoried data quality factors including availability of electronic health records, measurement of vital signs, registration of diagnostic tests, treatment and disposition, and availability of guidelines. Both the local factors as quality factors are described in a previous publication [17] and are summarized in Supplemental file 1. We explored the most influencing hospital-level factors (Total number of ED visits, supervision, availability of point-of care test (streptococcal antigen test and C-reactive protein), primary care during out-of-office hours) whether they significantly contributed to the adjusted model for antibiotic prescription. Antibiotic treatment was registered in the medical record. There were no differences in completeness of data between ambulatory and admitted patients (see Table 1). We agree more information regarding these EDs should be added in the Methods section.
- Data collection comprised data of routine care. The clinical teams were trained in clinical assessment and standard data registration in the health record for febrile children. The local research team entered data from healthcare records in the electronic case record form (eCRF) of MOFICHE. Data were not automatically derived from electronic health records. Every 3 months a data quality report including proportion of missing variables was communicated with the local research team in order to improve completeness of the collected data. Difficult cases were discusses locally or at monthly teleconferences. Differences in missing data were mainly influenced by local protocols. For example, in some EDs all vital signs (heart rate, respiratory rate, temperature, oxygen saturation) were measured routinely in all patients as in other EDs vital signs were measured at the discretion of the nurse or physician.

We have clarified these issues in the Methods section:

*“The EDs were included because they all participated in the PERFORM project. Characteristics of these EDs are described in S1 Appendix and in a previous publication [17]. In short, the participating hospitals were either university (n=9) or large teaching hospitals (n=3), and 11 EDs have paediatric paediatric intensive care facilities. Nine EDs were paediatric focused and three EDs served both children and adults. In five EDs, all data was available in electronic health care records, one ED used paper records and six EDs used a combination of paper and electronic health care records.”*

*“The local research team entered data from patient records in an electronic case record form (eCRF)”*

Results:

*“In addition, there were no differences in completeness of outcomes and diagnosis between ambulatory and admitted patients.”*

**3.2)**	What are provider-related factors that influence prescription between sites (type of provider, level of training)? Please represent in your data analysis.

**Answer 3.2:** We evaluated setting factors that varied across EDs and were related to antibiotic prescription according to the literature. Due to limited number of EDs, we were able to evaluate five hospital-level variables: total number of ED visits, supervision during out-of-office hours, availability of point-of-care tests (streptococcal antigen test and CRP) and primary care during out-of-office hours. Unfortunately, we do not have patient level data of background or experience of physician. To clarify, we have added this analysis in the results section of the paper and included the lack of patient level data on background or experience of physician as limitation in the discussion section.

Results:

*“None of the hospital-level factors was related with antibiotic prescription (range p value 0.14 -0.77).”*

Discussion:

*“Second, although experience of the physician (resident or consultant) and clinician specialty are related with antibiotic prescription [22, 23], we could not adjust for physician background at patient-level. However, we evaluated the contribution of supervision on antibiotic prescription on the hospital-level.* In our study, supervision was not related with antibiotic prescription.*”*

**3.3)**	I imagine that antibiotic prescription is largely related to local/national guidelines. For example, nitrofurantoine is specifically not recommended as first line for UTIs in some countries to spare the antibiotic; and TMP/SULFA is recommended as a first line instead. How was this assessed in the study? Could you add an analysis: per guideline/ not per guideline?

**Answer 3.3:** We thank the reviewer for this suggestion and added an analysis of prescription per guideline for respiratory tract infections and urinary tract infections. To compare the antibiotic prescription in our population with the guidelines, we analysed oral prescriptions in previous healthy children, who did not receive antibiotic treatment before ED visit. We have added this analysis in the Methods and Results section of the paper. We refer to answer 1.3 and 1.4 from reviewer 1.

**3.4)**	What are health-system related factors beyond guidelines that drive antibiotic prescription? Which ones were assessed/ not assessed?

**Answer 3.4:** We evaluated five hospital-level variables: total number of ED visits, supervision during out-of-office hours, availability of point-of-care tests (streptococcal antigen test and CRP) and primary care during out-of-office hours. See also answer 3.2 for change in results and discussion.

**3.5)**	I understand how the classification of antibiotics into narrow versus broad was reached; however, it is certainly disputable in some instances (e.g. pipercillin =narrow, cefuroxim=broad). Could you provide some

**Answer 3.5:** It is certainly disputable to define piperacillin as narrow. Since no established definition exists for broad-spectrum antibiotics and narrow-spectrum in ED studies, we searched literature for definitions of broad-spectrum vs narrow-spectrum and agreed in consensus meeting (PEFORM partners) to establish the final classification. Cefuroxime, however, is consistently classified as broad-spectrum antibiotic [2, 3, 24, 25]. For piperacillin, conflicting definitions are used. We chose to classify piperacillin in group ‘extended spectrum penicillins (e.g. amoxicillin) (J01CA)’ following the Anatomical Therapeutic Chemical classification. Since piperacillin was only prescribed in 18 patients (<1%) this will not influence our results.

**3.6)**	Furthermore, a better sense of the generalizability of the data should be provided. How was the site selection performed? How does it relate to national averages in key institutional/ health system aspects?

**Answer 3.6:** These EDs were included because they all participated in the PERFORM project which is a consortium in paediatrics infectious diseases. These EDs were all situated in university or large teaching hospitals, the 11 out of 12 had an onsite PICU and therefore these provide a high level of care. Generalization of these results to smaller hospital or on a national level should be done with caution.

This is mentioned in the Discussion section:

*“First, the included EDs are not representative for all febrile children attending the ED in that country. The EDs participating in this study are university or large teaching centres with ICU facilities involved in paediatric infectious disease research collaborations. Fever and sepsis guidelines were available in all EDs [17]. Therefore, these EDs represent a high standard of care and generalization of our findings to smaller hospitals or to a regional or national level, should be undertaken with caution.”*

We agree a better sense of generalization is important. We have performed a thorough literature and web search to identify data on European antibiotic prescription rates at emergency care. The European Centre for Disease Prevention and Control (ECDC) provides estimates of national antibiotic use in the community care and hospital care. However, data for the ED or hospital out-patients settings are not provided. In our opinion, due to major differences in setting, it is not possible to extrapolate national estimates of antibiotic prescription from community or hospital care to emergency care.

**3.7)** Are you looking at use or prescriptions? Two different concepts that are measured differently. Please clarify across the manuscript.

**Answer 3.7:** We are looking at antibiotic prescriptions and have clarified this across the manuscript.

Additional Minor Comments

**3.8)**

*	L113: the IQR appears out of context here. Specify

*	Introduction: give short introduction to PERFORM project

*	AIM: reformulate aim to align with your analysis. I think you would want to say: variations in rate and types of antibiotic prescription

**Answer 3.8:** We thank the reviewer for these comments to improve the introduction.

We have specified IQR (interquartile range), gave an introduction to the PERFORM project and adjusted the aim (see also answer 8 to comment 8 from editors).

*Introduction:*

*“In this study, we aim to investigate variation and appropriateness in rates and types of antibiotic prescription in febrile children attending 12 different ED’s in Europe. This is a main analysis of the MOFICHE study (Management and Outcome of Febrile children), which is embedded in the PERFORM project (Personalized Risk assessment in Febrile illness to Optimize Real-life Management across the European Union) [6]. MOFICHE is an observational multicentre study which studies the management and outcome of febrile children in Europe using routine data. The overall aim of PERFORM is to improve management of febrile children and to improve diagnosis through development of new diagnostic tests to discriminate viral and bacterial infections in children.”*

Methods

**3.9)**	How were the participating hospitals selected?

**Answer 3.9:** These EDs were included because they all participated in the PERFORM project which is a consortium in paediatrics infectious diseases. These EDs were all situated in university or large teaching hospitals, and 10 out of 12 had an onsite PICU. We added this in the Methods section:

*“Twelve EDs from eight European countries participated in this study: Austria, Germany, Greece, Latvia, the Netherlands (n=3), Spain, Slovenia, and the United Kingdom (n=3). These EDs were included because they all participated in the PERFORM project.”*

**3.10)**	Are these pediatric EDs? Who sees patients? GPs? Pediatricians? Emergency Medicine Specialists?

**Answer 3.10:** We agree that more information on the EDs is required. Nine EDs were paediatric focused EDs. The supervising specialist is a general paediatrician in 6 EDs, a (paediatric) emergency physician in 3 EDs and general paediatrician or (paediatric) emergency physician in 2 EDs. This is added in the Methods section:

*“Care for febrile children was supervised by general paediatricians (6 EDs), paediatric emergency physicians (2 EDs) or by a general paediatrician or a (paediatric) emergency physician (3 EDs).”*

**3.11)**	How was duration of data collection determined?

**Answer 3.11:** Duration of data collection was determined to be one year to reflect the seasonal variation. This is added in the Methods:

*“Data were collected from January 2017 until April 2018 for at least one year to include all seasons.”*

**3.12)**	The rationale for your sample size calculation is unclear to me; please expand. Comment on generalizability

**Answer 3.12**: We agree with the reviewer that the current sample size considerations are confusing. The sample size was estimated to be sufficient for the other aims of MOFICHE including other outcomes as hospitalization and blood culture rate. In the study protocol the sample size estimation was defined as follows: ‘We expect to include nearly 40,000 children with at most 5% of missing data. Pilot data indicate on overall antibiotic prescription rate of 30%, hospitalization rate of 10%, a CRP rate of 15% and a blood culture rate of 5%. Applying a commonly used rule of thumb of 10 events per variable, we expect that the study sample is large enough to analyse at least 120 determinants for the most prevalent outcome of antibiotic prescription and 25 determinants for the least prevalent outcome of blood culture with sufficient accuracy.’ We agree with the reviewer that the current sample size considerations are confusing. We have therefore adjusted the sample size considerations to estimate a desired with of a 95% confidence interval of the estimated standardized prescription rates. The number of ED visits varied between 400-7000 with an event rate of approximately 30%. These numbers would allow the expected width of the confidence interval of the standardized prescription rates to be below 0.5 for the smallest ED. We have clarified this in the Methods section:

Methods:

*“We expected to include 40,000 children with at most 5% of missing data. Pilot data showed an overall antibiotic prescription rate of 30%. Applying 10 events per variable, this study is large enough to analyse over 1000 determinants for the outcome antibiotic prescription. We performed a post-hoc sample size estimation for a desired width of the 95% confidence interval (CI) of standardized prescription rates. The expected width of the CI of the standardized prescription rates was below 0.5 for the smallest ED.”*

For our comment on generalizability we refer to answer 3.6.

**3.13)**	Does the missing at random assumption really hold here (for example various NICE traffic light factors are related amongst each other)? How did you decide on this?

**Answer 3.13:** Missing at random (MAR) occurs when missingness is not random, but where missingness can be fully accounted for by other variables in the database.. We included a wide ranges of variables in the imputation model, including all variables used in the adjusted model (general characteristics, NICE warning signs, vital signs, focus of infection and final diagnosis), but also other auxiliary variables (see the Statistical Analysis Plan). We evaluated the proportion of missing values stratified for each ED, for hospitalization (yes/no), referral (yes/no), triage urgency (high/low) and for groups of focus of infection. We found no clear differences in proportion of missing values between hospitalized patients and patients who were discharged home (table 1). Increased missingness for vital signs was related to self-referral and low triage urgency (table 23). Furthermore, missingness of NICE alarming signs was related to specific infections: in lower respiratory infections missingness for work of breathing was low (7%), but was higher in gastro-intestinal infections (18%) (table 4). Therefore, we performed multiple imputation under the MAR assumption.

**3.14)**	Did you exclude patients with missing data on antibiotic use or antibiotic prescription?

**Answer 3.14:** Yes, we excluded patients with missing data on antibiotic use, missing presumed cause of infection and focus of infection (n=738). This is mentioned in the Data analysis section and we have added analysis in the Results section and in a supplemental file comparing patients with complete outcome and patients with missing outcome:

*Methods:*

*“We excluded patients with missing data on antibiotic prescription, presumed cause of infection and focus of infection.”*

*Results:*

*“Of the total population of 38,480 patients, we excluded 738 patients based on missing data of antibiotics or diagnosis, and 2,092 patients with a repeated visit to the same ED. Compared to patients with complete outcome data, patients with missing data were similar in age, sex, comorbidity and admission rates (S6 appendix).”*

**3.15)**	How did you account for the difference in triage systems to account for "triage level"?

**Answer 3.15:** For the triage systems, 9 EDs used Manchester Triage System (MTS) and 3 used a local triage system based on the MTS. We merged the triage categories ‘immediate’, ‘very urgent’ and ‘urgent’ in high urgency and the categories ‘standard’ and ‘non-urgent’ in low urgency. We have clarified this in the methods section.

“Patient-level factors were selected a-priori and included general characteristics (age, sex, season, comorbidity, referral (referred vs self-referred), markers for disease severity *as triage urgency (high urgency (immediate, very urgent, urgent)* *versus low urgency* *(standard, non-urgent)),* …”

**3.16)***	"Second, we used multilevel logistic regression using clustering on hospital to study variation of antibiotic use between hospitals". This is unclear to me: if you introduce hospital as a random effect (clustering), you would not be able to compare between hospitals?

**Answer 3.16:** Adding hospital as a random intercept allows us to quantify the variation in antibiotic prescription rates between hospitals through the median odds ratio (MOR) which depends on the variance parameter of the random intercept. The MOR quantifies the median odds in prescription rates between two EDs, and allows for a direct comparison in effect size with other predictors of antibiotic prescription.

In the calculation of the standardized prescription rates we calculated the probability of antibiotic prescription assuming that the patient would show up at the average ED, and hence the difference between EDs did not impact the expected antibiotic prescriptions.

We agree this needs more clarification and have changed the methods section of the paper. We refer to answer 2.2 to reviewer 2 for changes in the Methods section.

**3.17)**	How did you decide on the CRP cutoffs? Based on what?

**Answer 3.17:** CRP cut-offs were based on cut-offs used in the flowchart to classify bacterial or viral aetiology (60 mg/L) [26] and on literature (20 mg/L) [7, 8]. The CRP cut-off of 20mg/L identified patients at low-risk of serious bacterial infection in a systematic review[7, 8] and the CRP cut-off of 60 mg/L identified patients at high-risk of serious bacterial infections. We have added these references in the Methods section.

“We investigated diagnostics, including CRP (not performed, <20, 20-60, >60 mg/L) *[7, 8, 26],…”*

Results

**3.18)**	Table 1: the range of patients classified under the triage category seems very wide. How is this explained?

**Answer 3.18:** The wide range of triage categories is explained by differences in case-mix of the different EDs. Some EDs only see referred patients (99%), who in general also have a higher triage urgency whilst other EDs see mainly self-referred patients with primary care problems who in general have a lower triage urgency.

**3.19)**	L 359: remained…. Is there a word missing?

Answer 3.19: We have clarified this sentence:

“*After adjustment of general characteristics, disease severity, CRP and cause of infection, variability remained in antibiotic prescription rates for patients with lower respiratory tract infection”*

**3.20)**	Fog 5/6: heat map of what?

Answer 3.20: Figure 5 represents heat map of standardized antibiotic prescription rates whilst Figure 6 represents heat map of standardized broad-spectrum vs narrow-spectrum rates per ED. We have added a legend for the heat maps to clarify this:

**“Fig 6. Heat map of standardized antibiotic prescription rates per ED for lower and upper respiratory tract infections.** All adjusted for age, sex, season, comorbidity, fever duration, alarming signs, CRP, cause of infection. *EDs were ordered according to standardized antibiotic prescribing rates, from low to high on the left vertical axis. The coloured box represents rank of standardized rates for each ED; red colours indicate rates >1, blue colours indicate rates <1 and rates equal to 1 are coloured white.”*

**“Fig 7. Heat map of standardized broad-spectrum vs narrow-spectrum rates per ED for lower and upper respiratory tract infections.** All adjusted for age, sex, season, comorbidity, fever duration, alarming, CRP, cause of infection, previous antibiotic use. *EDs were ordered according to standardized broad-spectrum prescribing rates, from low to high on the left vertical axis. The coloured box represents rank of standardized rates for each ED; red colours indicate rates >1, blue colours indicate rates <1 and rates equal to 1 are coloured white.”*

Any attachments provided with reviews can be seen via the following link:

# References

1.	van de Voort EMF, Mintegi S, Gervaix A, Moll HA, Oostenbrink R. Antibiotic Use in Febrile Children Presenting to the Emergency Department: A Systematic Review. Front Pediatr. 2018;6:260. PubMed PMID: 30349814.

2.	van de Maat J, van de Voort E, Mintegi S, Gervaix A, Nieboer D, Moll H, et al. Antibiotic prescription for febrile children in European emergency departments: a cross-sectional, observational study. Lancet Infect Dis. 2019. PubMed PMID: 30827808.

3.	Hersh AL, Shapiro DJ, Pavia AT, Shah SS. Antibiotic prescribing in ambulatory pediatrics in the United States. Pediatrics. 2011;128(6):1053-61. PubMed PMID: 22065263.

4.	Hicks LA, Bartoces MG, Roberts RM, Suda KJ, Hunkler RJ, Taylor TH, Jr., et al. US outpatient antibiotic prescribing variation according to geography, patient population, and provider specialty in 2011. Clin Infect Dis. 2015;60(9):1308-16. PubMed PMID: 25747410.

5.	Fleming-Dutra KE, Hersh AL, Shapiro DJ, Bartoces M, Enns EA, File TM, Jr., et al. Prevalence of Inappropriate Antibiotic Prescriptions Among US Ambulatory Care Visits, 2010-2011. JAMA. 2016;315(17):1864-73. PubMed PMID: 27139059.

6.	PERFORM consortium. Personalised Risk assessment in Febrile illness to Optimise Real-life Management (PERFORM): PERFORM; 2019 [cited 2019 10-09-2019]. Available from: <https://www.perform2020.org/>.

7.	Van den Bruel A, Thompson MJ, Haj-Hassan T, Stevens R, Moll H, Lakhanpaul M, et al. Diagnostic value of laboratory tests in identifying serious infections in febrile children: systematic review. BMJ. 2011;342:d3082. PubMed PMID: 21653621.

8.	Verbakel JY, Lemiengre MB, De Burghgraeve T, De Sutter A, Aertgeerts B, Bullens DMA, et al. Point-of-care C reactive protein to identify serious infection in acutely ill children presenting to hospital: prospective cohort study. Arch Dis Child. 2018;103(5):420-6. PubMed PMID: 29269559.

9.	Nijman RG, Vergouwe Y, Thompson M, van Veen M, van Meurs AH, van der Lei J, et al. Clinical prediction model to aid emergency doctors managing febrile children at risk of serious bacterial infections: diagnostic study. Bmj. 2013;346:f1706. PubMed PMID: 23550046.

10.	Galetto-Lacour A, Zamora SA, Gervaix A. Bedside procalcitonin and C-reactive protein tests in children with fever without localizing signs of infection seen in a referral center. Pediatrics. 2003;112(5):1054-60. PubMed PMID: 14595045.

11.	National Institute for Health and Care Excellence (NICE). Antimicrobial prescribing guidelines 2020 [cited 2020 17-02-2020]. Available from: <https://www.nice.org.uk/about/what-we-do/our-programmes/nice-guidance/antimicrobial-prescribing-guidelines>.

12.	Public Health England. Managing common infections: guidance for primary care 2019 [updated 22-11-2019; cited 2020 17-02-2020]. Available from: <https://www.gov.uk/government/publications/managing-common-infections-guidance-for-primary-care>.

13.	Pouwels KB, Hopkins S, Llewelyn MJ, Walker AS, McNulty CA, Robotham JV. Duration of antibiotic treatment for common infections in English primary care: cross sectional analysis and comparison with guidelines. Bmj. 2019;364:l440. PubMed PMID: 30814052.

14.	Hersh AL, Fleming-Dutra KE, Shapiro DJ, Hyun DY, Hicks LA, for the Outpatient Antibiotic Use Target-Setting W. Frequency of First-line Antibiotic Selection Among US Ambulatory Care Visits for Otitis Media, Sinusitis, and Pharyngitis. JAMA Internal Medicine. 2016;176(12):1870-2. doi: 10.1001/jamainternmed.2016.6625.

15.	Palms DL, Hicks LA, Bartoces M, Hersh AL, Zetts R, Hyun DY, et al. First-Line Antibiotic Selection in Outpatient Settings. Antimicrob Agents Chemother. 2019;63(11). PubMed PMID: 31548186.

16.	Poole NM, Shapiro DJ, Fleming-Dutra KE, Hicks LA, Hersh AL, Kronman MP. Antibiotic Prescribing for Children in United States Emergency Departments: 2009-2014. Pediatrics. 2019;143(2). PubMed PMID: 30622156.

17.	Borensztajn D, Yeung S, Hagedoorn NN, Balode A, von Both U, Carrol ED, et al. Diversity in the emergency care for febrile children in Europe: a questionnaire study. BMJ paediatrics open. 2019;3(1):e000456-e. doi: 10.1136/bmjpo-2019-000456. PubMed PMID: 31338429.

18.	Craig JC, Williams GJ, Jones M, Codarini M, Macaskill P, Hayen A, et al. The accuracy of clinical symptoms and signs for the diagnosis of serious bacterial infection in young febrile children: prospective cohort study of 15 781 febrile illnesses. Bmj. 2010;340:c1594. PubMed PMID: 20406860.

19.	Van den Bruel A, Haj-Hassan T, Thompson M, Buntinx F, Mant D, European Research Network on Recognising Serious Infection i. Diagnostic value of clinical features at presentation to identify serious infection in children in developed countries: a systematic review. Lancet. 2010;375(9717):834-45. PubMed PMID: 20132979.

20.	The National Institute for Health and Care Excellence (NICE). Fever in under 5s: assessment and initial managment CG160 May 2013 2013 [updated August 2017]. Available from: <https://www.nice.org.uk/guidance/cg160>.

21.	Galetto-Lacour A, Zamora SA, Andreola B, Bressan S, Lacroix L, Da Dalt L, et al. Validation of a laboratory risk index score for the identification of severe bacterial infection in children with fever without source. Arch Dis Child. 2010;95(12):968-73. PubMed PMID: 20515973.

22.	Frost HM, McLean HQ, Chow BDW. Variability in Antibiotic Prescribing for Upper Respiratory Illnesses by Provider Specialty. J Pediatr. 2018;203:76-85 e8. PubMed PMID: 30195553.

23.	Agiro A, Gautam S, Wall E, Hackell J, Helm M, Barron J, et al. Variation in Outpatient Antibiotic Dispensing for Respiratory Infections in Children by Clinician Specialty and Treatment Setting. Pediatr Infect Dis J. 2018;37(12):1248-54. PubMed PMID: 30408006.

24.	Sarpong EM, Miller GE. Narrow- and Broad-Spectrum Antibiotic Use among U.S. Children. Health Serv Res. 2015;50(3):830-46. PubMed PMID: 25424240.

25.	Aabenhus R, Siersma V, Hansen MP, Bjerrum L. Antibiotic prescribing in Danish general practice 2004-13. J Antimicrob Chemother. 2016;71(8):2286-94. PubMed PMID: 27107098.

26.	Herberg JA, Kaforou M, Wright VJ, Shailes H, Eleftherohorinou H, Hoggart CJ, et al. Diagnostic Test Accuracy of a 2-Transcript Host RNA Signature for Discriminating Bacterial vs Viral Infection in Febrile Children. Jama. 2016;316(8):835-45. PubMed PMID: 27552617.

# Tables

| **Table 1: Proportion of missing data according to hospital admission after ED visit** | **Non-hospitalised patients (n=28588)** | **Hospitalised patients (n=9892)** |
| --- | --- | --- |
|  | N (%) missing | N (%) missing |
| **Sex** | 1 (0.0) | 0 |
| **Comorbidity** | 268 (0.9) | 102 (1.0) |
| **Triage urgency** | 716 (2.5) | 460 (4.7) |
| **Referral** | 960 (3.4) | 205 (2.1) |
| **Fever duration in days** | 1936 (6.8) | 839 (8.5) |
| **Vital signs** |  |  |
| Temperature | 1669 (5.8) | 477 (4.8) |
| Heart rate | 2919 (10.2) | 588 (5.9) |
| Respiratory rate | 6614 (23.1) | 2179 (22.0) |
| Oxygen saturation | 4551 (15.9) | 1030 (10.4) |
| Capillary refill time | 2779 (9.7) | 1652 (16.7) |
| **NICE “red traffic lights” (alarming signs)** |  |  |
| Ill appearance | 901 (3.2) | 820 (8.3) |
| Work of breathing | 3420 (12.0) | 1453 (14.7) |
| Dehydration | 4814 (16.8) | 2163 (21.9) |
| Rash | 2994 (10.5) | 1419 (14.3) |
| Consciousness | 220 (0.8) | 170 (1.7) |
| Meningeal signs | 1153 (4.0) | 891 (9.0) |
| Focal neurology | 1337 (4.7) | 1120 (11.3) |
| Status epilepticus | 585 (2.1) | 556 (5.6) |
| **Therapeutic antibiotics use in last 7 days** | 561 (1.9) | 214 (2.2) |
| **Antibiotics prescribed** | 314 (1.1) | 170 (1.7) |
| **Antibiotic administration** | 113 (1.5) | 84 (1.8) |
| **Focus of infection** | 24 (0.1) | 3 (0.0) |
| **Final diagnosis** | 256 (0.9) | 16 (0.2) |

| **Table 2: Proportion of missing data according to referral status** | **Self-referred, n=21239** | **Referred, n=16076** |
| --- | --- | --- |
|  | N (%) missing | N (%) missing |
| **Comorbidity** | 206 (0.9) | 152 (0.9) |
| **Triage urgency** | 550 (2.6) | 559 (3.5) |
| **Fever duration in days** | 1601 (7.5) | 1092 (6.8) |
| **Vital signs** |  |  |
| Temperature | 1483 (6.9) | 644 (4.0) |
| Heart rate | 2512 (11.8) | 920 (5.7) |
| Respiratory rate | 5040 (23.7) | 3260 (20.3) |
| Oxygen saturation | 3414 (16.1) | 1399 (8.7) |
| Capillary refill time | 1167 (5.5) | 3123 (19.4) |
| **NICE “red traffic lights” (alarming signs)** |  |  |
| Ill appearance | 823 (3.9) | 833 (5.2) |
| Work of breathing | 1847 (8.7) | 2966 (18.4) |
| Dehydration | 3250 (15.3) | 3590 (22.3) |
| Rash | 1927 (9.1) | 2365 (14.7) |
| Consciousness | 162 (0.8) | 214 (1.3) |
| Meningeal signs | 772 (3.6) | 1198 (7.5) |
| Focal neurology | 781 (3.7) | 1638 (10.2) |
| Status epilepticus | 102 (0.5) | 1021 (6.4) |

| **Table 3: Proportion of missing data according to triage urgency** | **Low urgent, n=24085** | **High urgent , n=13219** |
| --- | --- | --- |
|  | N missing | N missing |
| **Comorbidity** | 195 (0.8) | 160 (1.2) |
| **Referral** | 728 (3.0) | 370 (2.8) |
| **Fever duration in days** | 1598 (6.6) | 954 (7.2) |
| **Vital signs** |  |  |
| Temperature | 1600 (6.6) | 304 (2.3) |
| Heart rate | 2656 (11.0) | 557 (4.2) |
| Respiratory rate | 6558 (27.2) | 1581 (12.0) |
| Oxygen saturation | 4094 (17.0) | 1080 (8.2) |
| Capillary refill time | 2845 (11.8) | 1332 (10.1) |
| **NICE “red traffic lights” (alarming signs)** |  |  |
| Ill appearance | 785 (3.3) | 798 (6.0) |
| Work of breathing | 2360 (9.8) | 2374 (18.0) |
| Dehydration | 2498 (10.4) | 4244 (32.1) |
| Rash | 1667 (6.9) | 2581 (19.5) |
| Consciousness | 149 (0.6) | 195 (1.5) |
| Meningeal signs | 1106 (4.6) | 872 (6.6) |
| Focal neurology | 1644 (6.8) | 761 (5.8) |
| Status epilepticus | 990 (4.1) | 132 (0.9) |

| **Table 4: Proportion of missing data according focus diagnosis** | **URTI, n=20119** | **LRTI, n=5652** | **Gastro-intestinal, n=3988** | **Urinary tract, n=1353** | **Undifferentiated fever, n=2995** | **Other, n=4346** |
| --- | --- | --- | --- | --- | --- | --- |
|  | N (%) missing | N (%) missing | N (%) missing | N (%) missing | N (%) missing | N (%) missing |
| **Comorbidity** | 155 (0.8) | 67 (1.2) | 41 (1.0) | 14 (1.0) | 20 (0.7) | 73 (1.7) |
| **Referral** | 728 (3.0) | 370 (2.8) | 692 (3.4) | 92 (1.6) | 99 (2.5) | 162 (3.7) |
| **Triage urgency** | 485 (2.4) | 196 (3.5) | 110 (2.8) | 73 (5.4) | 88 (2.9) | 224 (5.2) |
| **Fever duration in days** | 1101 (5.5) | 519 (9.2) | 380 (9.5) | 97 (7.2) | 189 (6.3) | 488 (11.2) |
| **Vital signs** |  |  |  |  |  |  |
| Temperature | 1146 (5.7) | 301 (5.3) | 171 (4.3) | 68 (5.0) | 201 (6.7) | 248 (5.7) |
| Heart rate | 1975 (9.8) | 308 (5.4) | 294 (7.4) | 114 (8.4) | 384 (12.8) | 419 (9.6) |
| Respiratory rate | 4611 (22.9) | 966 (17.1) | 971 (24.3) | 315 (23.3) | 752 (25.1) | 1165 (26.8) |
| Oxygen saturation | 3036 (15.0) | 358 (6.3) | 584 (14.6) | 223 (16.5) | 610 (20.4) | 756 (17.4) |
| Capillary refill time | 1877 (9.3) | 658 (11.6) | 512 (12.8) | 208 (15.4) | 413 (13.8) | 752 (17.3) |
| **NICE “red traffic lights” (alarming signs)** |  |  |  |  |  |  |
| Ill appearance | 604 (3.0) | 362 (6.4) | 178 (4.5) | 83 (6.1) | 133 (4.4) | 349 (8.0) |
| Work of breathing | 2338 (11.6) | 397 (7.0) | 725 (18.2) | 227 (16.8) | 468 (15.6) | 707 (16.3) |
| Dehydration | 3188 (15.8) | 1222 (21.6) | 567 (14.2) | 273 (20.2) | 595 (19.9) | 1120 (25.8) |
| Rash: | 1936 (9.6) | 925 (16.4) | 586 (14.7) | 195 (14.4) | 314 (10.5) | 445 (10.2) |
| Consciousness | 132 (0.7) | 67 (1.2) | 42 (1.1) | 15 (1.1) | 39 (1.3) | 84 (1.9) |
| Meningeal signs | 699 (3.5) | 376 (6.7) | 250 (6.3) | 116 (8.6) | 152 (5.1) | 439 (10.1) |
| Focal neurology | 932 (4.6) | 463 (8.2) | 293 (7.3) | 141 (10.4) | 180 (6.0) | 436 (10.0) |
| Status epilepticus | 481 (2.4) | 203 (3.6) | 143 (3.6) | 50 (3.7) | 113 (3.8) | 151 (3.5) |
