# Supplementary material for: Variation in antibiotic prescription rates in febrile children presenting to emergency departments across Europe (MOFICHE): A multicentre observational study
Source: PLoS Med. 2020 Aug 19;17(8):e1003208. doi: 10.1371/journal.pmed.1003208 (PMC7444592; doi:10.1371/journal.pmed.1003208)
Supplement: Attachment — Submitted filename: 20200703_Replyreviewers_rev2_final.docx [file pmed.1003208.s016.docx]

Dear Dr. Moll,

Thank you very much for re-submitting your manuscript "Variation in antibiotic prescription rates in febrile children presenting to Emergency Departments across Europe (PERFORM): a multicentre observational study" (PMEDICINE-D-19-04008R1) for review by PLOS Medicine.

I have discussed the paper with my colleagues and the academic editor and it was also seen again by two reviewers. I am pleased to say that provided the remaining editorial and production issues are dealt with we are planning to accept the paper for publication in the journal.

The remaining issues that need to be addressed are listed at the end of this email. Any accompanying reviewer attachments can be seen via the link below. Please take these into account before resubmitting your manuscript:
------------------------------------------------------------
Dear Thomas McBride,

We are very pleased to hear that our manuscript is planned to be accepted in PLOS Medicine. We have improved our manuscript according to the recommendations of the editors and reviewers.

Please find below our responses to the minor issues raised by the editors and reviewers and the production issues.

Requests from Editors and Reviewers:
***1.1**- Thank you for agreeing to make your data available in a public data repository. At this time, please provide the url of the data repository, the DOI for the dataset, and any other information needed for readers to access the dataset (including the data contact email already provided).

Answer 1.1: The URL of the dataset and the codebook: [https://data.hpc.imperial.ac.uk/resolve/?doi=7251](https://eur01.safelinks.protection.outlook.com/?url=https%3A%2F%2Fdata.hpc.imperial.ac.uk%2Fresolve%2F%3Fdoi%3D7251&data=02%7C01%7Cn.hagedoorn%40erasmusmc.nl%7C15c7a557aa7143b8409d08d812d2d6e8%7C526638ba6af34b0fa532a1a511f4ac80%7C0%7C0%7C637280044046451782&sdata=nznimTXFBLIuUen3yA%2BlNGx7mhrDrEkz0mDQjxFZXnk%3D&reserved=0). DOI: 10.14469/hpc/7251

This is added in the data availablility statement.

“A data set containing individual participant data is available in a public data repository containing a specific DOI: https://data.hpc.imperial.ac.uk/resolve/?doi=7251. DOI: 10.14469/hpc/7251

The data is anonymized and does not contain any identifiable data. The data manager of the PERFORM consortium can be contacted for inquiries (Tisham.de08@imperial.ac.uk).”

***1.2**- Thank you for editing your title. Is it more appropriate to include MOFICHE than PERFORM, though?

Answer 1.2: We agree MOFICHE is more appropriate and changed the title according this suggestion.

Title: “Variation in antibiotic prescription rates in febrile children presenting to Emergency Departments across Europe (*MOFICHE*): a multicentre observational study”

***1.3**- The url for PERFORM can be removed from the Abstract and supplied in the Introduction or Methods.

Answer 1.3: We removed the url from the Abstract and supplied it in the Introduction.

Introduction: *“*This is a main analysis of the MOFICHE study (Management and Outcome of Febrile children), which is embedded in the PERFORM project (Personalized Risk assessment in Febrile illness to Optimize Real-life Management across the European Union, [*www.perform2020.org*](http://www.perform2020.org) *)”*

***1.4**- In the Abstract Methods and Findings section, please include the overall antibiotic prescription rate (all hospitals combined) along with the ranges. Please also specify that the ranges are across the different hospitals.

*Answer 1.4: We have added the overall antibiotic prescription rate and specified that the ranges are across the different EDs in the Abstract (Methods and Findings section).

Abstract, Methods and findings:

“In this analysis of 35,650 children (median age 2.8 years, 55% male), overall antibiotic prescription rate was *31.9% (range EDs:* 22.4-41.6%), and among those broad-spectrum antibiotics rate *was 52.1%* (*range EDs:* 33.0-90.3%).”

***1.5**- Thank you for providing more details in the Abstract Conclusions. This section could be a bit more succinct. For example, the second and third sentences are redundant given the previous section.
***1.6**- Please edit the last sentence of the Abstract Conclusions to read: “Until better diagnostics are available to accurately differentiate between bacterial and viral aetiologies, implementation of antimicrobial stewardship guidelines across Europe is necessary to limit antimicrobial resistance.”

Answer 1.5 and 1.6:

Thank you for this excellent suggestion, we have changed the last section of Abstract Conclusions according to this recommendation and shortened this section.

Conclusions

“In this study, we observed wide variation between European EDs of prescriptions of antibiotics and broad-spectrum antibiotics in febrile children. ~~Although minor variation was observed in bacterial infection, great variation was present in viral infections and infections of unknown cause.~~ Overall, one third of prescriptions were inappropriate or inconclusive with marked variation between EDs. ~~In respiratory and urinary infections, guideline-concordance of prescriptions varied widely across EDs.~~ “*Until better diagnostics are available to accurately differentiate between bacterial and viral aetiologies, implementation of antimicrobial stewardship guidelines across Europe is necessary to limit antimicrobial resistance.”*

***1.7**- Thank you for adding an Author Summary. The second point can be removed.
Answer 1.7: We have removed the second point in the Author Summary:

*~~“Variation of antibiotic prescription and its relation with viral or bacterial disease is unknown for all children with fever at the ED~~*”

***1.8**- Additionally, please edit the “What Did the Researchers Do and Find?” section of the Author Summary could be edited for brevity, perhaps focusing on the standardized prescription rates and the prescriptions with inappropriate indication or duration.

Answer 1.8:

What did the researchers do and find?

“ -	In this prospective observational study, we included routine information of 35,650 children (median age 2.8 years) with fever attending 12 different EDs in Europe and calculated the proportion of antibiotic prescriptions and broad-spectrum antibiotic prescriptions. We adjusted for differences in population including age, comorbidity, disease severity, and focus and cause of infection.

-	Across EDs, antibiotic prescription rates ranged between 22.4-41.6%, and of these prescriptions, broad-spectrum antibiotics rates ranged between 33.0-90.3%. Standardized antibiotic prescription rates ranged between 0.77 and 1.35 and standardized rates of broad-spectrum antibiotics ranged between 0.65-1.75.

~~-	Standardized antibiotic rates varied in viral infections (0.1 to 3.3), infections of unknown cause (0.1 to 1.8). Reassuringly, in bacterial infections, antibiotic prescription was comparable between EDs (0.9 to 1.1).~~

-	Prescription which were inappropriately indicated ranged 0.6-29.3%, inconclusive prescriptions ranged 0.5-61.7%. Inappropriate duration of oral prescriptions ranged 4.4-59.0%. ~~Prescriptions which were not concordant to the guideline ranged 11.8-47.3% in uncomplicated respiratory tract infections and ranged 11.1-100% in uncomplicated urinary tract infections.”~~

***1.9**- Please also add “In this study we found…”, “These findings suggest...”, or similar to the “What Do These Findings Mean?” section of the Author Summary.

Answer 1.9: We have edited the Author Summary following this suggestion:

“*In this study we found* variation of prescription of antibiotics and broad-spectrum antibiotics between EDs in children with fever even corrected for age, comorbidity, disease severity, diagnostics, and focus and cause of infection.”

***1.10**- Thank you for noting ethics approval. Please provide a list (Supplemental Text is fine) of the ethics committees from the participating hospitals.
***1.11**- Please make reference to the S4 appendix (details of the regression model) in the main text Methods section.

***1.12-** S7 Appendix: typo in the title.

***1.13-** S12, S14 Appendix: Please make sure the title in the file matches the title in the list of supplemental files in the main text.

***1.14-** S15 Appendix is a file with tracked changes, please supply a clean version.

Answer 1.11: Thank you, we have adjusted the supplemental files according to these recommendations.

*Ethics approval in supplemental S3 Text.

*We mentioned the S7 appendix in the main text Methods section in the paragraph ‘Data analysis’.

“Specifications of the adjusted model are presented in table 1 and in *S7 Text.”*

*S9 Text:

*“Descriptive characteristics of cases with complete outcomes and cases with missing outcomes”*

**“S11 Fig. Range of antibiotic prescriptions (A) and broad-spectrum prescriptions (B) per emergency department (ED) for viral, bacterial and unknown bacterial/viral”*

*We have removed the tracked changes from S13 Text.

***1.15**- The data presented in Figure 2 would be better presented as a table. Please revise accordingly.
Answer 1.15: We have changed the data presented in Figure 2 as a table (table 4).

***1.16**- Please revise the stacked bar chart shown in Figure 3 as a side-by-side bar graph.
Answer 1.16: We have revised Figure 2 as a side-by-side bar graph. In the side-by-side bar graph the proportion of antibiotic prescriptions per ED is not as easy to compare across EDs and the overall antibiotic prescription is also not shown in the figure. This is better visualized in the stacked bar chart. We have uploaded both figures (Figure 2a and Figure 2b) and leave it to the Editor to decide which figure is most appropriate. [according to editorial comments from 1^st^ July, figure 2 is provided as stacked bar chart].

***1.17**- Figure 6: include Otitis media and Tonsillitis/pharyngitis in the title?
Answer 1.17: Following the suggestion by reviewer 1 (see answer 2.3) we have removed this analysis and figures from the paper. [according to editorial comments from 1^st^ July, the analysis for respiratory tract infections are added in the Supplemental. We included otitis media and tonsillitis/pharyngitis in the title of the supplemental].

Results:

*“Results of variation of antibiotic and broad-spectrum prescriptions for respiratory tract infections are provided in S11 Text.”*

*“S11 Text. Variation of antibiotic and broad-spectrum prescription in lower respiratory, otitis media, tonsillitis/pharyngitis and other upper respiratory tract infections.”*

***1.18**- Figures 4-7: it seems there is plenty of room to provide 95% CIs in these fgures without asking the reader to open a supplementary file. Please consider adding.
Answer 1.18: We agree and added the 95% CIs in these figures.

***1.19**- Figures 4-8: the legends are not much use without a scale for the gradient.
Answer 1.19: We agree and added a scale for the gradient for these figures.

***1.20**- Discussion, second paragraph: “Our study supports previous studies that reported variable antibiotic prescribing…”?
***1.21**- Discussion, final paragraph: “Until better diagnostics are available to accurately differentiate between bacterial and viral aetiologies, we strongly urge the implementation of antimicrobial stewardship guidelines to reduce antibiotic prescription in febrile children across Europe.”

Answer 1.20 and 1.21: Thanks for these suggestions and have adjusted these sentences:

*“Our study supports previous studies that reported variable antibiotic prescribing for all febrile children..”*

*“Until better diagnostics are available to accurately differentiate between bacterial and viral aetiologies, we strongly urge the implementation of antimicrobial stewardship guidelines to reduce antibiotic prescription in febrile children across Europe.”*

1.22- Please move the mention of the analysis plan and the STROBE checklist to earlier in the Methods so that they appear as S1 and S2.
Answer 1.22: We have changed the mentioning of the analysis plan and strobe checklist to the first paragraph of the Methods section.

*“This study is reported as per the Strengthening the Reporting of Observational Studies in Epidemiology (STROBE) guideline (S1 Text) and data were analysed using an a priori statistical analysis plan (S2 Text).”*

Comments from Reviewers:

Reviewer #1:

***2.1**) I appreciate the efforts that the authors have made to respond to the comments. I remain concerned, however, about the unclear implications of all the variation they document. The authors write in the introduction that "understanding variability is essential for the development and implementation of interventions to optimize antibiotic use." Why? They never explicitly state what the implications would be if they showed low variability versus high variability on each of their outcomes.

It appears that the five main outcomes are as follows: 1) the antibiotic prescribing rate; 2) the proportion of antibiotics that were broad-spectrum; 3) the % of antibiotics that were for appropriate (presumed bacterial), inappropriate (presumed viral) or of unclear appropriateness (viral or bacterial); 4) the % of antibiotics that were of inappropriate duration; and 5) the % of antibiotics for urinary tract infections and respiratory infections that were guideline-concordant. Here are my view of the implications of variation in each of these outcomes.

**-*2.1-A** Variability in overall antibiotic prescribing rate. Documenting variability in this rate is not that helpful in my view. The authors seem to implicitly believe that the variation implies overprescribing, but since the ideal rate of prescribing is unknown, it is unclear if the high prescribing EDs are overprescribing, if the low prescribing EDs is underprescribing, or both.

Answer 2.1-A:

We thank the reviewer for this thorough review and useful recommendations to improve the paper. We agree with the reviewer that the implications of variations need more emphasis and explanation in the introduction and the discussion.

The variation in antibiotic and broad-spectrum prescription suggest a certain degree of unnecessary practice variation. We reason this implies overprescribing since several studies at the EDs show that only 7 to 13% of febrile children has a serious bacterial infection which need antibiotic treatment. Second, respiratory tract infections are the most common infection in febrile children and overprescribing was shown in a previous study [1]. Therefore, variation far above these numbers of bacterial infections are overprescriptions. Furthermore, we adjusted for an extensive number of factors influencing disease severity and antibiotic prescribing including case-mix and markers of disease severity (age, comorbidity, fever duration, warming signs) and also focus and cause of infection. These analyses make the antibiotic rate comparable between EDs.

We have added this in the introduction and discussion:

Introduction:

“Fever is one of the most common reasons for children to visit the Emergency Department (ED) and *most visits are accounted for by self-limiting infections*[2, 3]. *Children with* a serious bacterial infection that needs treatment with antibiotics *range 7-13% while antibiotic rates in febrile children at EDs are between 19-64%* [1, 4, 5]. *Inappropriate antibiotic prescriptions were described in around 30% of outpatient prescriptions [6, 7].* However, these outpatient settings mainly involve primary care settings and limited studies are available on emergency care.”

Discussion:

“The diversity in antibiotic prescribing across different EDs appears not to be associated with antimicrobial resistance or immunization coverage. *Although the ideal antibiotic prescription rate is unknown, the diversity in antibiotic prescribing suggests overprescribing. Prescription rates were above the average incidence of serious bacterial infections. We found variation in antibiotic prescription rates, even when adjusted for general characteristics, disease severity, diagnostics and focus and cause of infection. This suggests room for improvement in reduction of antibiotic prescriptions and especially broad-spectrum prescriptions at the ED.*”

-***2.1-B** Variability in broad-spectrum prescribing - same as above. The ideal rate of broad-spectrum prescribing is unknown.

Answer 2.1-B:

For prescribing broad-spectrum antibiotics, it is known that narrow-spectrum antibiotics are first choice at the ED in general and have similar clinical outcomes and less adverse events. Therefore, we think our analysis suggest that variation in broad-spectrum prescription suggests a certain degree of unnecessary practice variation.

We have emphasized this argument in the discussion.

Discussion:

“The diversity in antibiotic prescribing across different EDs appears not to be associated with antimicrobial resistance or immunization coverage. *Although the ideal antibiotic prescription rate is unknown, this diversity in antibiotic prescribing suggests overprescribing. Prescription rates were above the average incidence of serious bacterial infections and we found variation in antibiotic prescription rates, even when adjusted for general characteristics, disease severity, diagnostics and focus and cause of infection.* *This suggests room for improvement in reduction of antibiotic prescriptions and especially broad-spectrum prescriptions at the ED.* EDs with higher antibiotic prescription rates did not necessarily prescribe more broad-spectrum antibiotics. The ED with highest standardized broad-spectrum rate (UK, 3), did not have high inappropriate prescriptions for respiratory tract infections. In only this ED, amoxicillin-clavulanate (broad-spectrum) was first-choice agent for uncomplicated respiratory tract infections which could explain higher broad-spectrum rates in this ED. Studies demonstrated that use of narrow-spectrum antibiotics compared to broad-spectrum antibiotics leads to similar clinical outcomes and to fewer adverse events [8, 9]. Unnecessary use of broad-spectrum antibiotics potentially increases resistance rates even further.

- ***2.1-C** Variability in prescribing for antibiotic-inappropriate conditions (e.g., viruses) - documenting variability is helpful because the ideal rate of use is known (0%). If there is a lot of variation with some very high prescribing EDs, it would imply that some EDs that are doing way worse than others and therefore quality improvement initiatives should perhaps take a more targeted approach focused on the outliers. If there is little variation and the rate of prescribing among EDs is high, that would imply a more global approach is needed. If there is little variation and the rate of prescribing among EDs is consistently closer to 0%, then maybe we don't need to worry about overprescribing for viral infections.
Note that I don't feel the same about prescribing for bacterial infections - how would finding variation inform stewardship initiatives? I'm not sure it would, so it's not clear this analysis needs to be included. Also, variability in prescribing for unknown bacterial/viral infection is hard to interpret because the ideal rate of prescribing is unknown, so I'm also not sure it needs to be included.

Answer 2.1-C:

Our analysis show that variation in antibiotic prescribing increases with increasing diagnostic uncertainty. For bacterial infections, we found little variation whereas for unknown bacterial/viral infections, variation was larger. We agree that the ideal rate is unknown in the group of unknown bacterial/viral infections. In order to interpret the variation in viral infections, we think it useful to also show variation in bacterial infections and unknown bacterial/viral infections. In our study, we observed that EDs with higher standardized prescription rates for viral infections also have higher standardized prescription for unknown bacterial/viral infections, even after adjustment for case-mix, disease severity and focus of infection. We agree this needs more explanation in the manuscript.

Discussion:

*“In addition, diversity of antibiotic prescription increased with diagnostic uncertainty. After adjustment of general characteristics, disease severity, diagnostics and focus of infection,* we observed *minor variation in bacterial infections, and larger* variability of antibiotic prescriptions in viral infections and unknown bacterial/viral infections *In general, EDs with higher antibiotic prescriptions in viral infections also had higher antibiotic prescriptions in unknown bacterial/viral infections. This indicates that overprescribing in viral infections is linked to higher prescriptions in unknown bacterial/viral infections. Diagnostic uncertainty in patients with an unclear cause of infection could be reduced by improved targeted antibiotic prescription from new diagnostic signatures of bacterial and viral infection.*”

-***2.1-D** Variability in prescribing of inappropriate duration - this is helpful because we know the rate should be 0%

- Variability in guideline concordance - this is helpful because we know the rate should be 100%.

I would urge the authors to more explicitly walk the reader through the implications of variation in each outcome in the Discussion, using the above as points to consider incorporating. Also, in the introduction, it would be helpful to motivate the analysis by arguing that assessing the degree of variation on outcomes that should be 0% or 100% can inform whether quality improvement initiatives should be global versus targeted.

Answer 2.1-D):

We have adjusted the introduction and discussion to emphasize the implications of variation:

Introduction:

“A better understanding of variability in antibiotic prescriptions in EDs and its relation with bacterial or viral disease, taking into account differences in case-mix, is essential for the development and implementation of interventions to optimize antibiotic use*. In addition, knowledge regarding variation of prescribing in infections where antibiotic prescription is inappropriate such as prescriptions in viral disease, prescriptions of inappropriate duration, or prescriptions that are not concordant to guidelines, could target and improve implementation of antimicrobial stewardship guidelines at ED level.*“

Discussion:

“The diversity in antibiotic prescribing across different EDs appears not to be associated with antimicrobial resistance or immunization coverage. *Although the ideal antibiotic prescription rate is unknown, this diversity in antibiotic prescribing suggests overprescribing*. Prescription rates were above the average incidence of serious bacterial infections and we found variation in antibiotic prescription rates, even when adjusted for general characteristics, disease severity, diagnostics and focus and cause of infection. *This suggests room for improvement in reduction of antibiotic prescriptions and especially broad-spectrum prescriptions at the ED.* EDs with higher antibiotic prescription rates did not necessarily prescribe more broad-spectrum antibiotics. (..)

*“In addition, diversity of antibiotic prescription increased with diagnostic uncertainty. After adjustment of general characteristics, disease severity, diagnostics and focus of infection,* we *observed minor variation in bacterial infections, and larger variability of antibiotic prescriptions in viral infections and unknown bacterial/viral infections. In general, EDs with higher antibiotic prescriptions in viral infections also had higher antibiotic prescriptions in unknown bacterial/viral infections. This indicates that overprescribing in viral infections is linked to higher prescriptions in unknown bacterial/viral infections. Diagnostic uncertainty in patients with an unclear cause of infection could be reduced by improved targeted antibiotic prescription from new diagnostic signatures of bacterial and viral infection.”* (..)

“*We evaluated appropriateness in indication, duration and guideline-concordance. Ideally, EDs should target 100% of appropriateness in these three aspects of antibiotic prescribing.* In our study, we did not observe a clear association between inappropriate indicated prescriptions and prescriptions of inappropriate duration. This indicates that guideline implementations should focus on these different aspects of appropriate antibiotic prescribing*. Furthermore, quality improvement initiatives should be emphasized on EDs with higher proportions of inappropriate prescriptions. In addition,* future antimicrobial stewardship interventions across Europe should focus on reducing broad-spectrum treatment and antibiotic use in viral infections.”

Presentation issues

***2.2** Abstract is hard to follow. For example, it reports guideline-concordant prescribing but does not mention earlier that this will be an outcome. It comes out of nowhere.

I would restructure as follows. State that the main outcomes are 1) the antibiotic prescribing rate; 2) the proportion of antibiotics that were broad-spectrum; 3) the % of antibiotics that were for appropriate (presumed bacterial), inappropriate (presumed viral) or of unclear appropriateness (viral or bacterial); 4) the % of antibiotics that were of inappropriate duration; and 5) the % of antibiotics for urinary tract infections and respiratory infections that were guideline-concordant. Then state that the overall rate across all children was calculated. Then state that for each outcome, variation was assessed by calculating standardized prescribing rates (i.e., the ratio between observed and expected antibiotic prescribing rate) using multilevel logistic regression models. Then in the results, report the overall result and the range in standardized prescription rates for each of the five outcomes.

Answer 2.2:

We agree the different outcomes need to be more clearly presented in the abstract. We agree it is helpful for the reader to structure as suggested: 1) the antibiotic prescribing rate; 2) the proportion of antibiotics that were broad-spectrum; 3) the % of antibiotics that were for appropriate (presumed bacterial), inappropriate (presumed viral) or of unclear appropriateness (viral or bacterial); 4) the % of antibiotics that were of inappropriate duration; and 5) the % of antibiotics for urinary tract infections and respiratory infections that were guideline-concordant.

Abstract Methods and Findings:

*“Main outcomes were 1) antibiotic prescription rate, 2) the proportion of antibiotics that were broad-spectrum antibiotics, 3) the proportion of antibiotics of appropriate indication(presumed bacterial), inappropriate indication (presumed viral) or inconclusive (unknown bacterial/viral), 4) the proportion of oral antibiotics of inappropriate duration, and 5) the proportion antibiotics that were guideline-concordant in uncomplicated urinary and upper and lower respiratory tract infections (RTI). We determined variation of antibiotic prescription and broad-spectrum prescription by calculating standardized prescription rates using multilevel logistic regression and adjusted for general characteristics (age, sex, comorbidity, referral), disease severity (triage level, fever duration, presence of alarming signs), use and result of diagnostics, and focus and cause of infection. In this analysis of 35,650 children (median age 2.8 years, 55% male), overall antibiotic prescription rate was 31.9% (range EDs: 22.4-41.6%), and among those broad-spectrum antibiotics rate was 52.1% (range EDs 33.0-90.3%).”*

***2.3** Abstract and discussion both state that antibiotic stewardship initiatives are needed until better diagnostics are available. That's simply not true. Even with perfect diagnostics, we would still need stewardship initiatives (e.g., to ensure appropriate selection and duration of antibiotics).

**Answer 2.3:** We agree with the reviewer that antimicrobial stewardship is of importance to guide appropriate antibiotic prescribing, also if better diagnostics are available.

We have adjusted the discussion to clarify this.

Discussion:

*“This indicates that guideline implementations should focus on these three different aspects of appropriate antibiotic prescribing* *to ensure prescriptions of appropriate indication, duration and antibiotic selection. Furthermore, quality improvement initiatives should be emphasized on EDs with higher proportions of inappropriate prescriptions. In addition, future antimicrobial stewardship interventions across Europe should focus on reducing broad-spectrum treatment and antibiotic use in viral infections.*

***2.4:** In general, the manuscript is also difficult to follow. There are a lot of analyses, and they are not always "signposted" well. It would be helpful to have a section in the Methods called "Outcomes" that defines each of the five main outcomes. Then have a section called "Statistical analysis" that describes the statistics used to assess the outcomes overall and to describe variation.

Answer 2.4:

We agree that the different outcomes and analyses need more clarification in the Methods and Results section of the manuscript. We have added a paragraph describing the different outcomes and positioned the outcomes more clearly in the data-analysis section. In addition, we have more clearly defined the subheadings in the results section of the manuscript and provided the overall rate and ranges consistently.

Methods:

*Outcomes*

*“We assessed various aspects of antibiotic prescription: 1) antibiotic prescription rate, 2) the proportion of antibiotics that were broad-spectrum vs narrow-spectrum and 3) the proportion of antibiotics of ‘likely appropriate’ indication (presumed bacterial), ‘likely inappropriate’ indication (presumed viral) or ‘inconclusive’ (unknown bacterial/viral), 4) the proportion of oral antibiotics of inappropriate duration, and 5) the proportion of oral antibiotics that matched the antibiotic type in the guideline (‘guideline-concordant’) in uncomplicated urinary and upper and lower respiratory tract infections (RTI). Antibiotic prescriptions were classified as ‘likely appropriate’ in presumed bacterial infections (definite bacterial, probable bacterial, bacterial syndrome), ‘likely inappropriate’ in prescriptions in presumed viral infections (definite viral, probable viral, viral syndrome), and ‘inconclusive’ in prescriptions in unknown bacterial/viral infections or others. Inappropriate duration was defined as >10 days for treatment of tonsillitis with beta-lactamase sensitive penicillins (J01CE) and >7 days for all other prescriptions according to recommendations by international guidelines [10-12]. In addition, ‘guideline-concordant prescription’ in patients with uncomplicated upper and lower respiratory tract infections and uncomplicated urinary tract infections, was defined according to the local guideline (S5 appendix). Uncomplicated infections were defined as infections in previously healthy children who did not receive therapeutic antibiotic treatment before ED visit.”*

Data analysis

(…)”First, we performed a descriptive analysis of the frequency of antibiotic prescription, broad-spectrum and narrow-spectrum prescription, including ranges across EDs. *For all outcomes, we calculated the overall proportion and proportion per ED. Second, we used multilevel logistic regression with a random intercept for each ED to study variation between EDs for antibiotic prescription, broad-spectrum prescription vs narrow-spectrum prescription, and intravenous/intramuscular vs oral prescriptions [13].*(….)

*Appropriateness of antibiotic prescriptions*

*“We calculated standardized rates for antibiotic prescription and broad-spectrum prescription in groups of presumed viral infections, presumed bacterial infections and unknown bacterial/viral infections. Next, we assessed the proportion of all antibiotic prescriptions that were ‘likely appropriate’, ‘likely inappropriate’ and ‘inconclusive. For all oral prescriptions, we calculated the proportion of prescriptions that were both inappropriate in indication (‘likely inappropriate’) and of inappropriate duration, and the proportion of prescriptions that were either inappropriate in indication or inappropriate in duration. In uncomplicated respiratory tract and urinary tract infections, we calculated the proportion of all oral prescriptions that were inappropriate for all the three estimates (indication, duration and guideline-concordance), and the proportion of prescriptions that were inappropriate in any of the three groups. R version 3.4 was used for the analysis and visualization of the data.”*

Results:

Subheadings:

*“Study population”*

*“Overall antibiotic prescriptions”*

*“Variation of overall antibiotic prescription and broad-spectrum prescription”*

*“Variation of antibiotic and broad-spectrum prescriptions in viral infections, bacterial infections and unknown/bacterial/viral infections”*

Antibiotic prescription rate was 6.9% (1418/20383) for viral infections (range EDs: 0.4-18.9%), 88.8% (1280/1451) for definite bacterial infections (range EDs: 83.5-96.2%), 94.7% (6097/6438) for presumed bacterial infections (range EDs: 81.2-99.3%) and 45.2% (2348/5200) for unknown bacterial/viral infections (range EDs: 1.7-79.3%) (S12 Appendix).

*“Variation in prescriptions of appropriate indication and appropriate duration”*

Of all antibiotic prescriptions, 65.0% (7386/11371) were estimated as likely appropriate (range EDs: 23.7-98.9%), 12.5% (1418/11371) were likely inappropriate (range EDs: 0.6-29.3%) and 22.6% (2567/11371) were inconclusive (range EDs: 0.5-61.7%).

Oral antibiotic prescriptions with inappropriate duration were found in 20.0% (1525/7636) and this ranged 4.4-59.0% across EDs. Of all oral antibiotic prescriptions, 2.1% (134/7636) were both of inappropriate indication and inappropriate duration (range EDs 0.0-8.4%), whereas 30.0% (2294/7636) were either of inappropriate indication or of inappropriate duration (range EDs: 11.3-69.9%) (Fig. 8).

*“Variation of appropriate prescriptions in uncomplicated respiratory tract infections and urinary tract infections”*

In uncomplicated respiratory tract infections, oral prescriptions were not guideline-concordant in 22.3% (973/4373) (*range EDs: 11.8-47.3%).* In this group, proportion of prescriptions that were inappropriate in all three estimates (indication, duration and guideline-concordance) was 0.7% (31/4373), whilst 42.3% (1850/4373) were inappropriate in any of the three estimates (*range EDs: 15.7-80.9%).* In uncomplicated urinary tract infections, oral prescriptions were not concordant to the guideline in 45.1% (152/337) (*range EDs:* 11.1-100%) and 65.9% (222/337) were inappropriate in any of the three estimates (*range EDs: 11.1-100%).*

***2.5** I don't think the antimicrobial resistance analysis is that helpful - it's distracting and comes out of nowhere in my view. Consider deleting.

Answer 2.5: We agree with the reviewer that there is too much emphasis on antimicrobial resistance in the paper. However, the antimicrobial resistance data is of importance to interpret variation in broad-spectrum prescription. We therefore have shortened the sections in the methods and results and provided the details in the Supplemental file.

**Methods:**

*“Next, since antimicrobial resistance patterns vary greatly between European countries, standardized rates of broad-spectrum vs narrow-spectrum antibiotics were compared with antimicrobial resistance data of invasive isolates on a national level and the local hospital [14, 15] (S8 Text).”*

**Results:**

*“Higher standardized rates for broad-spectrum antibiotics were not related to higher antimicrobial resistance percentages on a national level or on a hospital level (S8 Text).”*

***2.6** The prescribing for respiratory tract infections also seems a little bit extraneous, in large part because it's not clear what the ideal rate of prescribing should be for something like ear infections. Consider deleting. Readers will have an easier time digesting this very dense manuscript if there are just the five analyses for the five outcomes and if the analyses are reported in a consistent manner.

Answer 2.6: According to the editorial comments from 1^st^ July, the analysis covering the variation in antibiotic prescription and broad-spectrum rates in respiratory tract infections is mentioned in the supplemental information.

Results:

*“Results of variation of antibiotic and broad-spectrum prescriptions for respiratory tract infections are provided in S11 Text.”*

“S11 Text. Variation of antibiotic and broad-spectrum prescription in lower respiratory, otitis media, tonsillitis/pharyngitis and other upper respiratory tract infections.”

***3.1** Reviewer #2: The authors have conducted a very thorough job providing additional analyses and responding to my review comments. The manuscript, upon reading, is much clearer and strengthened. I have no additional points to raise.

Answer 3.2: We thank reviewer #2 for his/her previous comments and thank him/her for the positive conclusion that the manuscripts is much cleared and the additional analyses have strengthened the manuscript.

# Production and editorial issues, 1^st^ July 2020

Dear Jessica Hyde,

Thanks a lot for checking our manuscripts

As requested, we have changed the submission according to the recommendations below.

Best wishes,

Nienke Hagedoorn

EDITORIAL:

1. Thank you for providing the url and DOI associated with the dataset. Please update the data statement as follows (if accurate):
“
An anonymized data set containing individual participant data is available in a public data repository: <https://eur01.safelinks.protection.outlook.com/?url=https%3A%2F%2Fdata.hpc.imperial.ac.uk%2Fresolve%2F%3Fdoi%3D7251&amp;data=02%7C01%7Ch.a.moll%40erasmusmc.nl%7Cc2d88ca33dfd45ee8fe608d81de64a8e%7C526638ba6af34b0fa532a1a511f4ac80%7C0%7C0%7C637292222222491200&amp;sdata=urVI9KuSVZHgutirixvOTeQKQcMFD%2FHaPNZRH%2F8dD8o%3D&amp;reserved=0>. DOI: 10.14469/hpc/7251

For inquiries to obtain the full dataset, please contact the data manager of the PERFORM consortium (Tisham.de08@imperial.ac.uk).
“

Answer 1: We have adjusted the data statement as suggested:

“An anonymized data set containing individual participant data is available in a public data repository: https://data.hpc.imperial.ac.uk/resolve/?doi=7251. DOI: 10.14469/hpc/7251

For inquiries to obtain the full dataset, please contact the data manager of the PERFORM consortium (Tisham.de08@imperial.ac.uk)”

2. Please add “In this cohort…” or similar to the third point of the “What Do These Findings Mean?” section of the Author Summary.
**Answer 2:** We have added this in the Author Summary:

What Do These Findings Mean?

- *“In this cohort of febrile children,* one third of prescriptions were of inappropriate or inconclusive indication with variation between EDs. In addition, guideline-concordance for respiratory and urinary infections varied widely across EDs.”

3. Figure 2: you’re right, the stacked bar chart makes comparisons across EDs easier. Please present one graph (Fig 2 b). Apologies for this request.
**Answer 3:** We have presented 2b (the stacked bar chart) as figure 2.

4. Lines 980-983 (Marked up version): perhaps clearer to say: “After adjustment of general characteristics, disease severity, diagnostics and focus of infection, we observed minor variation in bacterial infections, and larger variability of antibiotic prescriptions in viral infections and unknown bacterial/viral infections. ”

**Answer 4:** We agree this sentence is clear and adjusted the sentence accordingly:

Discussion:

“*After adjustment of general characteristics, disease severity, diagnostics and focus of infection,* we observed minor variation in antibiotic prescribing in bacterial infections, and larger variability of antibiotic prescriptions in viral infections and *unknown bacterial/viral infections*.”

5. Though Reviewer #1 suggested the analysis of prescriptions for respiratory tract infections be removed, perhaps these could still be included as a supplemental text for interested readers. If you do add this back in, please include a brief mention of these results and reference to the supplemental text in the Results section.

**Answer 5:** We have added the analysis of prescriptions of respiratory tract infections as a supplemental and included a brief mention in the Results section.

**Results:**

“Results of variation of antibiotic and broad-spectrum prescriptions for respiratory tract infections are provided in S11 Text.”

S11 Text. Variation of antibiotic and broad-spectrum prescription in lower respiratory, otitis media, tonsillitis/pharyngitis and other upper respiratory tract infections.


FIGURES and GRAPHICS:

1. Figures 1, 3, 4, and 5 are acceptable for publication.
-Figure 2 is a multipanel figure. Multipanel figures (those with parts A, B, etc.) should be submitted as a single file that contains all parts of the figure. Label the figure itself with capital letters, Arial bold font, 12 points.

2. Please note that Figure 2 is currently split into multiple parts in separate files. Please either combine the parts into one figure with lettered panels, or renumber your figures as whole parts (i.e. Fig 2A becomes Fig 2, Fig 2B becomes Fig 3 etc.), ensuring that all relevant legends and in-text citations have been updated accordingly.

**Answer 1,2:** See editorial comment 3. We will remove Fig2a and add only Fig 2b. We adjusted this in the text and figure captions.

Results:

“Most prescribed broad-spectrum antibiotics were combinations of penicillins with beta-lactamase inhibitors (2309/11371, 20.3%), second-generation cephalosporins (1154/11371, 10.1%) and third-generation cephalosporins (1097/11371, 9.6% (Table 4, Fig 2).

“Fig 2. Antibiotic classes for prescribed antibiotics per ED, n=35650, Emergency Departments (EDs) are sorted on antibiotic prescription rate per ED. NL, the Netherlands; UK, United Kingdom.”

METADATA:

1. Please accurately complete the 'Competing Interests' section on our submission form, including any COIs declared by your co-authors. If you have no competing interests to declare, please add "The authors have declared that no competing interests exist". Otherwise please declare all competing interests beginning with the statement "I have read the journal's policy and the authors of this manuscript have the following competing interests:"
**Answer 1:**

We have adjusted the Competing Interests’ section on the submission form:

*“I* *have read the journal's policy and the authors of this manuscript have the following competing interests:* MvdF received a grant from CSL Behring preclinical work immunoglobulin formulations; FS served on the scientific advisory board for Roche on Lyme disease serological diagnostics, received research support from the Slovenian Research Agency [grant number P3-0296], and is an unpaid member of the steering committee of the ESCMID Study Group on Lyme Borreliosis/ESGBOR.

The other authors have declared that no competing interests exist.”

2. As your paper involves contributions from PERFORM consortium, please format your paper as follows:

1) Please list PERFORM consortium as an author in the byline.
2) If you wish to include the names of individual group members in the manuscript, you may list them in the Acknowledgments section.

Please be aware that group author members are no longer indexed in PubMed as collaborators.

**Answer 2:** We have added PERFORM consortium in the byline and also added PERFORM consortium as author in the submission form. The individual group members are listed in the supplemental file and do not need listing in the Acknowledgments section.

ARTICLE FILE:

1. Please indicate the corresponding author in the byline by placing an asterisk (*) after their affiliation number.

**Answer 1**: We have added an asterisk after the affiliation number of the corresponding author.

“….Henriette A Moll^1^*”

2. Please include the corresponding author's email address on the title page of your manuscript, indicated by an asterisk (*). Only an asterisk and the email address itself are required.
Answer 2: We included the correspondin autor’s email on the title page indicated by an asterisk.

“ * [h.a.moll@erasmusmc.nl](mailto:h.a.moll@erasmusmc.nl) “

3. We have noted that you have the following present in your manuscript: Abbreviations list/ List of keywords

We have removed these section from your manuscript file as this information is automatically pulled from the submission form. Please make sure that the information in the submission form is correct.
**Answer 3:** We have removed the keywords and abbreviations list form the article file.

REFERENCES:

1. Please put all reference citations in square brackets separated by either commas or dashes with no spaces, e.g. [1,2,3] or [1-3].

2. Please make sure all references are cited in ascending numerical order in the text.
**Answer 1 and 2:** We have checked all the reference citations.


Production issues 3rd of June

FIGURES and GRAPHICS:

1. Figure 1 is low resolution and will appear blurry or pixelated if published. Text and images should appear crisp and clear. Please note that this problem will not be detected or corrected by the PACE tool, and this will not be fixed by simply increasing the resolution of your Figures in the image editing software. Please carefully review the PLoS Figure Guidelines and provide Figure files that were originally created at 300 pixels/inch (dpi) and at least 6.68cm wide, but less than 19.05cm and under 22.225cm tall. For further information, please see the Figure Guidelines (<https://eur01.safelinks.protection.outlook.com/?url=http%3A%2F%2Fjournals.plos.org%2Fplosmedicine%2Fs%2Ffigures&amp;data=02%7C01%7Ch.a.moll%40erasmusmc.nl%7C400b84eb40ad4e473b9408d80801f428%7C526638ba6af34b0fa532a1a511f4ac80%7C0%7C0%7C637268151773241205&amp;sdata=HUgxwZFypP%2FxaXrC99fJY3UGofpA7XKfw%2Fkf26n7BBg%3D&amp;reserved=0>).

We exported figure 1 in high resolution.

2. Figures 2, 4, 5, 6, and 7 are acceptable for publication.

Perfect.

3. Figures 3 and 8 currently contain more than ½ an inch of white space on any side. Please remove unnecessary white space, leaving about ⅛ inch of white space outside of the image. Once you have made your selection, go to Image → Crop to Selection. After cropping, always be sure to check the size.

Yes, we cropped figures 2 and figure 5, and checked the size and used PACE to adjust the files to .tiff.

4. Please ensure that all main figure files are cited in ascending numerical order in the main text of the article.

Yes, all figure files are cited in ascending numerical order.

5. Please ensure that all main figures are referenced in the manuscript as Fig 1, Fig 2, etc. (including capitalization), rather than Figure 1, Figure 2, etc. Please note, however, that the file names themselves must not include the space, i.e. Fig1.tif, Fig2.tif, etc.

All main figures are referenced as needed and the file names are adjusted.

For Figure 2 we uploaded two figure options (Figure 2A and Figure 2B) and we leave it up to the editor to decide which figure is most appropriate.

6. Please ensure that all main figure legends are placed after the paragraph containing their first in-text citation.

Yes, all figure legends are placed after the paragraph containing the first in-text citation.

TABLES:

1. Please ensure that all main tables in their entirety (including titles and footnotes), are placed after the paragraph containing their first in-text citation.

Yes, checked.

2. Table 1 uses returns to control text wrapping. This is indicated by a [¶ or ↵] symbol when "Show Paragraph Marks" is turned on in Word. Please either split those cells into separate cells to achieve alignment or remove the returns.

Yes, we corrected this in all tables.

METADATA:

1. Please accurately complete the 'Competing Interests' section on our submission form, including any COIs declared by your co-authors. If you have no competing interests to declare, please add "The authors have declared that no competing interests exist". Otherwise please declare all competing interests beginning with the statement "I have read the journal's policy and the authors of this manuscript have the following competing interests:"

2. Your co-author, Dr. van der Flier, has declared the following competing interest in relation to this manuscript. Please include it in the main competing interest statement in our system, sticking as closely as possible to Dr. van der Flier's wording, using only their initials.

"Grant CSL Behring preclinical work immunoglobulin formulations"

3. Your co-author, Dr. Strle, has declared the following competing interest in relation to this manuscript. Please include it in the main competing interest statement in our system, sticking as closely as possible to Dr. Strle's wording, using only their initials.

"I served on the scientific advisory board for Roche on Lyme disease serological diagnostics, received research support from the Slovenian Research Agency [grant number P3-0296], and am an unpaid member of the steering committee of the ESCMID Study Group on Lyme Borreliosis/ESGBOR. The funders had no role in the design of the study; in the collection, analyses, or interpretation of data; in the writing of the manuscript, or in the decision to publish the results."

4. Please ensure only the authors' initials are included in the competing interest statement, not their full names or titles.

Our competing interest statement is formulated as followed:

“MvdF received a grant from CSL Behring preclinical work immunoglobulin formulations. FS served on the scientific advisory board for Roche on Lyme disease serological diagnostics, received research support from the Slovenian Research Agency [grant number P3-0296], and is an unpaid member of the steering committee of the ESCMID Study Group on Lyme Borreliosis/ESGBOR. The other authors have declared that no competing interests exist.”

5. Please ensure that the funders and grant numbers match between the Financial Disclosure field and the Funding Information tab in your submission form. Note that the funders must be provided in the same order in both places as well.

This project has received funding from the European Union’s Horizon 2020 research and innovation programme under grant agreement No. 668303. The Research was supported by the National Institute for Health Research Biomedical Research Centres at Imperial College London, Newcastle Hospitals NHS Foundation Trust and Newcastle University. The views expressed are those of the author(s) and not necessarily those of the NHS, the NIHR or the Department of Health. For the remaining authors no sources of funding were declared.

The funders had no role in study design, data collection and analysis, decision to publish, or preparation of the manuscript.

6. In accordance with PLOS' data policy, please ensure that your Data Availability Statement in the submission form clearly identifies how readers can access your data. Data cannot be available on request, or only accessible by contacting one of the authors. Note that your Data Availability Statement will be typeset as it is written, so please ensure it is in complete sentences and appears as you would like it to in the published version.

For details on our data policy, please see <https://eur01.safelinks.protection.outlook.com/?url=http%3A%2F%2Fjournals.plos.org%2Fplosmedicine%2Fs%2Fdata-availability&amp;data=02%7C01%7Ch.a.moll%40erasmusmc.nl%7C400b84eb40ad4e473b9408d80801f428%7C526638ba6af34b0fa532a1a511f4ac80%7C0%7C0%7C637268151773251161&amp;sdata=R2ozuf1q5bgGc13nCbGSQcRymUojQgP05pgll%2BHsD%2FQ%3D&amp;reserved=0>

7. We note that your data has been deposited into a public repository. Please include the name(s) of repositories, as well as DOIs or accession numbers for the relevant data sets in your Data Availability Statement. An example is “All relevant data are within the paper and its Supporting Information files except for the sequence of XXX which is available from Genbank under the accession number XXX”.

Our data statement is formulated as followed:

“A data set containing individual participant data is available in a public data repository containing a specific DOI: https://data.hpc.imperial.ac.uk/resolve/?doi=7251. DOI: 10.14469/hpc/7251

The data is anonymized and does not contain any identifiable data. The data manager of the PERFORM consortium can be contacted for inquiries (Tisham.de08@imperial.ac.uk).”

ARTICLE FILE:

1. Please indicate the corresponding author in the byline by placing an asterisk (*) after their affiliation number.

2. Please include the corresponding author's email address on the title page of your manuscript, indicated by an asterisk (*). Only an asterisk and the email address itself are required.

This is adjusted.

3. The name for your co-author Nienke N Hagedoorn does not match across the article file and the submission form. Please ensure that all author names match verbatim, including the use of a middle name/initials and any special characters (accents, umlauts, etc.), as correct spelling and formatting of author names is vital for accurate indexing in PubMed.

This is adjusted.

4. Please remove the funding information from your manuscript file. The published version is pulled automatically from the “Financial Disclosure” field in the submission form and must not be duplicated. Please therefore take this opportunity to check that the information in this field is correct and completed in full.

Funding information is removed from manuscript file.

5. Please ensure that your Ethics statement is available in the Methods section of your manuscript in its entirety.

Ethics statement is available in the methods section and in detail in the supplemental (S3 Text) .

REFERENCES:

1. Please ensure your references are in the style of PLOS Medicine (ICMJE/Vancouver style). The reference style guide can be found here: <https://eur01.safelinks.protection.outlook.com/?url=http%3A%2F%2Fjournals.plos.org%2Fplosmedicine%2Fs%2Fsubmission-guidelines%23loc-references&amp;data=02%7C01%7Ch.a.moll%40erasmusmc.nl%7C400b84eb40ad4e473b9408d80801f428%7C526638ba6af34b0fa532a1a511f4ac80%7C0%7C0%7C637268151773251161&amp;sdata=CLeUq6OvCt1rv71TzgAH0Hg5IOOtd%2Be07DIuYc5qsSg%3D&amp;reserved=0>. You can also find a downloadable EndNote file in these guidelines to assist you. Please do a manual check as well, as the EndNote file does not catch everything. Please note that the references will be typeset as they appear so please ensure the format is consistent.

2. Please put all reference citations in square brackets separated by either commas or dashes with no spaces, e.g. [1,2,3] or [1-3].

3. Please make sure all references are cited in ascending numerical order in the text.

We checked al l the references for being in the right format.

SUPPORTING INFORMATION:

1. Please ensure that all Supporting Information files use the following format verbatim throughout the manuscript (including legends and all in-text citations): S1 Fig, S1 Table, S1 Text, S1 Data, etc. Each file type should start at 1 and increase numerically. Please note, however, that the file names themselves must use an underscore rather than a space, i.e. S1_Fig.tif, S1_Table.xlsx, S1_Text.doc, S1_Data.xlsx, S1_PRISMA_Checklist.docx, etc.

Yes, we have checked the numbering and referencing of the supporting information.

2. Please ensure that each supporting information file has a legend at the end of the manuscript file, after the Reference list.

Yes, legend is listed after the reference list.

3. Please note that your submission cannot contain any references to generic "supporting information" files and instead must refer to specific files using our naming convention (S1 Fig, S1 Table, S1 Text, S1 Data, S1 PRISMA Checklist, etc.)

Yes, we changed the numbering.

4. Please upload your supporting information as individual files, and use the following naming format: S1 Text; S1 Fig; S1 Table, S1 Data, etc. Each file type should start as S1 and increase numerically: S1 Text, S2 Text, etc. Note that all relevant in-text citations and captions will need to be updated accordingly.

Yes, we checked the in-text citations and captions.

If you'd prefer to keep your supporting information in one file, please edit any citation of embedded figures and/or tables within your Supporting Information files and the main manuscript to use letters rather than numbers, e.g. “Table A in S1 Text” or "Fig B in S1 Appendix.".

We provided separate files for the supporting information.

**References**

1.	van de Maat J, van de Voort E, Mintegi S, Gervaix A, Nieboer D, Moll H, et al. Antibiotic prescription for febrile children in European emergency departments: a cross-sectional, observational study. Lancet Infect Dis. 2019. PubMed PMID: 30827808.

2.	Poropat F, Heinz P, Barbi E, Ventura A. Comparison of two European paediatric emergency departments: does primary care organisation influence emergency attendance? Ital J Pediatr. 2017;43(1):29. PubMed PMID: 28274237.

3.	Sands R, Shanmugavadivel D, Stephenson T, Wood D. Medical problems presenting to paediatric emergency departments: 10 years on. Emerg Med J. 2012;29(5):379-82. PubMed PMID: 21609944.

4.	Craig JC, Williams GJ, Jones M, Codarini M, Macaskill P, Hayen A, et al. The accuracy of clinical symptoms and signs for the diagnosis of serious bacterial infection in young febrile children: prospective cohort study of 15 781 febrile illnesses. BMJ. 2010;340:c1594. PubMed PMID: 20406860.

5.	Nijman RG, Vergouwe Y, Thompson M, van Veen M, van Meurs AH, van der Lei J, et al. Clinical prediction model to aid emergency doctors managing febrile children at risk of serious bacterial infections: diagnostic study. BMJ. 2013;346:f1706. PubMed PMID: 23550046.

6.	Fleming-Dutra KE, Hersh AL, Shapiro DJ, Bartoces M, Enns EA, File TM, Jr., et al. Prevalence of Inappropriate Antibiotic Prescriptions Among US Ambulatory Care Visits, 2010-2011. JAMA. 2016;315(17):1864-73. PubMed PMID: 27139059.

7.	Hersh AL, Shapiro DJ, Pavia AT, Shah SS. Antibiotic prescribing in ambulatory pediatrics in the United States. Pediatrics. 2011;128(6):1053-61. PubMed PMID: 22065263.

8.	Gerber JS, Ross RK, Bryan M, Localio AR, Szymczak JE, Wasserman R, et al. Association of Broad- vs Narrow-Spectrum Antibiotics With Treatment Failure, Adverse Events, and Quality of Life in Children With Acute Respiratory Tract Infections. JAMA. 2017;318(23):2325-36. PubMed PMID: 29260224.

9.	Williams DJ, Hall M, Shah SS, Parikh K, Tyler A, Neuman MI, et al. Narrow vs broad-spectrum antimicrobial therapy for children hospitalized with pneumonia. Pediatrics. 2013;132(5):e1141-8. PubMed PMID: 24167170.

10.	National Institute for Health and Care Excellence (NICE). Antimicrobial prescribing guidelines 2020 [cited 2020 17-02-2020]. Available from: <https://www.nice.org.uk/about/what-we-do/our-programmes/nice-guidance/antimicrobial-prescribing-guidelines>.

11.	Public Health England. Managing common infections: guidance for primary care 2019 [updated 22-11-2019; cited 2020 17-02-2020]. Available from: <https://www.gov.uk/government/publications/managing-common-infections-guidance-for-primary-care>.

12.	Pouwels KB, Hopkins S, Llewelyn MJ, Walker AS, McNulty CA, Robotham JV. Duration of antibiotic treatment for common infections in English primary care: cross sectional analysis and comparison with guidelines. BMJ. 2019;364:l440. PubMed PMID: 30814052.

13.	Bates D, Maechler M, Bolker B, Walker S. Fitting Linear Mixed-Effects Models Using lme4. Journal of Statistical Software. 2015;67:1-48. doi: 10.18637/jss.v067.i01.

14.	European Centre for Disease Prevention and Control. Surveillance of antimicrobial resistance in Europe - Annual report of the European Antimicrobial Resistance Surveillance Network (EARS-Net): European Centre for Disease Prevention and Control; 2018 [cited 2019 15-03-2019]. Available from: <https://ecdc.europa.eu/sites/portal/files/documents/EARS-Net-report-2017-update-jan-2019.pdf>.

15.	World Health Organization. Prioritization of pathogens to guide discovery, research and development of new antibiotics for drug-resistant bacterial infections, including tuberculosis. : World Health Organization; 2017 [cited 2019 25-03-2019]. Available from: <https://www.who.int/medicines/areas/rational_use/PPLreport_2017_09_19.pdf?ua=1>.
